# Fluorite and rare-metals mineralization in greisenized granite of Um Naggat area, Egypt constrained by physical separation and beneficiation assessment

**DOI:** 10.1038/s41598-026-58961-6

**Published:** 2026-07-01

**Authors:** Mona M. Fawzy, Mohamed Diab, Ahmed E. Abdel Gawad

**Affiliations:** Nuclear Materials Authority, El-Maadi, P.O. Box 530, Cairo, Egypt

**Keywords:** Fluorite, Rare metals mineralization, Physical separation, Greisenized granites, Um Naggat area, Egypt, Environmental sciences, Solid Earth sciences

## Abstract

Fluorite and rare-metals mineralization hosted within the greisenized granites of Um Naggat area, Central Eastern Desert, Egypt, represents a potential source of critical raw materials. The studied sample is dominated by quartz and feldspar, with a minor heavy-mineral fraction enriched in fluorite and accessory rare-metal phases, including zircon, columbite, ilmenite, cassiterite, uranothorite, monazite, aegirine, almandine garnet, iron oxides, and biotite. Comprehensive mineralogical, geochemical, and beneficiation investigations were conducted to assess the recovery potential of fluorite and associated rare-metals mineralization. Particle-size analysis and mineralogical characterization indicated favorable liberation of the target minerals, enabling efficient separation from the silicate gangue. Gravity concentration using a Wilfley shaking table upgraded the total heavy-mineral (THM) content from 1.95 wt% in the feed to 6.11 wt% in the combined concentrate, achieving an overall THM recovery of ~ 80%. Subsequent high-intensity magnetic separation effectively partitioned the concentrate into fluorite-rich diamagnetic and rare-metals-bearing magnetic fractions. XRD and SEM-EDS analyses indicate that fluorite is the dominant phase in the diamagnetic product, accompanied by accessory zircon and galena, while columbite, thorite, garnet, and other rare-metals-bearing minerals are concentrated in the magnetic fraction. The results demonstrate the effectiveness of a combined gravity–magnetic beneficiation flowsheet for upgrading fluorite and associated rare-metals mineralization from the studied greisenized granite under laboratory-scale conditions. These findings provide a basis for further process optimization and detailed technical and economic evaluation of Um Naggat mineralization.

## Introduction

Fluorite (CaF₂) is a strategically important industrial mineral and the principal source of fluorine for global chemical production. Fluorine is primarily extracted from fluorite through the manufacture of hydrofluoric acid (HF), which serves as a key intermediate in the production of numerous fluorine-based compounds, including fluoropolymers, fluorocarbons, aluminum fluoride (AlF₃), and cryolite (Na₃AlF₆), all of which are essential for the chemical, metallurgical, and aluminum industries^1–3^. In addition, HF is used in the production of uranium hexafluoride (UF₆) for nuclear fuel processing, as well as in specialty applications such as optics, ceramics, and glass manufacturing^4^. More recently, fluorine-bearing compounds have gained increasing importance in advanced energy technologies, particularly in lithium-ion batteries, where they enhance performance, stability, and lifespan^5^.

Among the various geological settings, greisen-hosted systems represent an important source of fluorite and associated rare metals^6–8^. Greisenization is a hydrothermal alteration process resulting from fluid–rock interactions, characterized by the replacement of primary minerals such as feldspars and biotite by quartz and white mica, often accompanied by fluorite and topaz. This process is closely linked to the formation of rare-metals mineralization enriched in elements such as Zr, Nb, Ta, Sn, W, Li, and rare earth elements (REEs)^9,10^. The evolution of greisen systems is strongly controlled by the composition of hydrothermal fluids, particularly fluorine concentration and pH, while processes such as fluid boiling can enhance fluorine activity and promote mineralization.

Despite their economic importance, fluorite-bearing greisen systems are commonly characterized by complex mineralogical assemblages in which fluorite is closely associated with silicate gangue and a variety of accessory minerals. Such mineralogical complexity can hinder efficient mineral recovery and necessitates the development of suitable beneficiation strategies. Although froth flotation is the conventional industrial technique for fluorite beneficiation, its effectiveness generally relies on fine grinding to achieve adequate mineral liberation, which increases energy consumption, reagent usage, and overall processing costs. In contrast, physical separation methods, including gravity and magnetic separation, provide efficient pre-concentration and upgrading routes where sufficient contrasts in specific gravity and magnetic susceptibility exist among constituent minerals^11–16^. These techniques are particularly suitable for ores exhibiting relatively coarse-grained and well-liberated mineral phases, offering a cost-effective and environmentally favorable alternative to chemically intensive beneficiation processes^17–19^. In Um Naggat greisenized granites, fluorite occurs predominantly as coarse and relatively well-liberated grains, making gravity and high-intensity magnetic separation technically appropriate and economically attractive options for mineral recovery and upgrading.

In Egypt, the granitic rocks of Um Naggat area in the Central Eastern Desert have attracted considerable research interest due to their enrichment in fluorite and strategic rare metals^20–24^. These granites represent a significant source of economically important elements, including Zr, Nb, Ta, Sn, W, F, Y, U, Th, and REEs, which are widely recognized as critical “high-tech” metals due to their essential roles in modern technologies such as renewable energy systems, electric vehicles, advanced electronics, and high-performance materials^25–28^.

However, despite previous mineralogical and geochemical investigations, limited attention has been given to the systematic evaluation of the beneficiation behavior of fluorite and associated minerals in Um Naggat greisenized granites. In particular, there is a lack of quantitative assessment of physical separation efficiency in terms of grade, recovery, and concentrate quality.

Accordingly, the main objective of this study is to characterize the mineralogical features and quantitatively evaluate the physical beneficiation potential of the greisenized granites from Um Naggat area, Central Eastern Desert, Egypt. This evaluation is based on measurable parameters, including total heavy mineral (THM) grade, recovery efficiency, concentrate purity, and liberation assessment based on particle-size distribution and mineralogical examination. Furthermore, the study aims to assess the performance of eco-friendly physical separation techniques, specifically gravity concentration (shaking table) and high-intensity magnetic separation, in enhancing the recovery and upgrading of valuable mineral phases, and to evaluate the overall economic potential of these greisenized rocks.

## Geologic setting

**Fig. 1:**
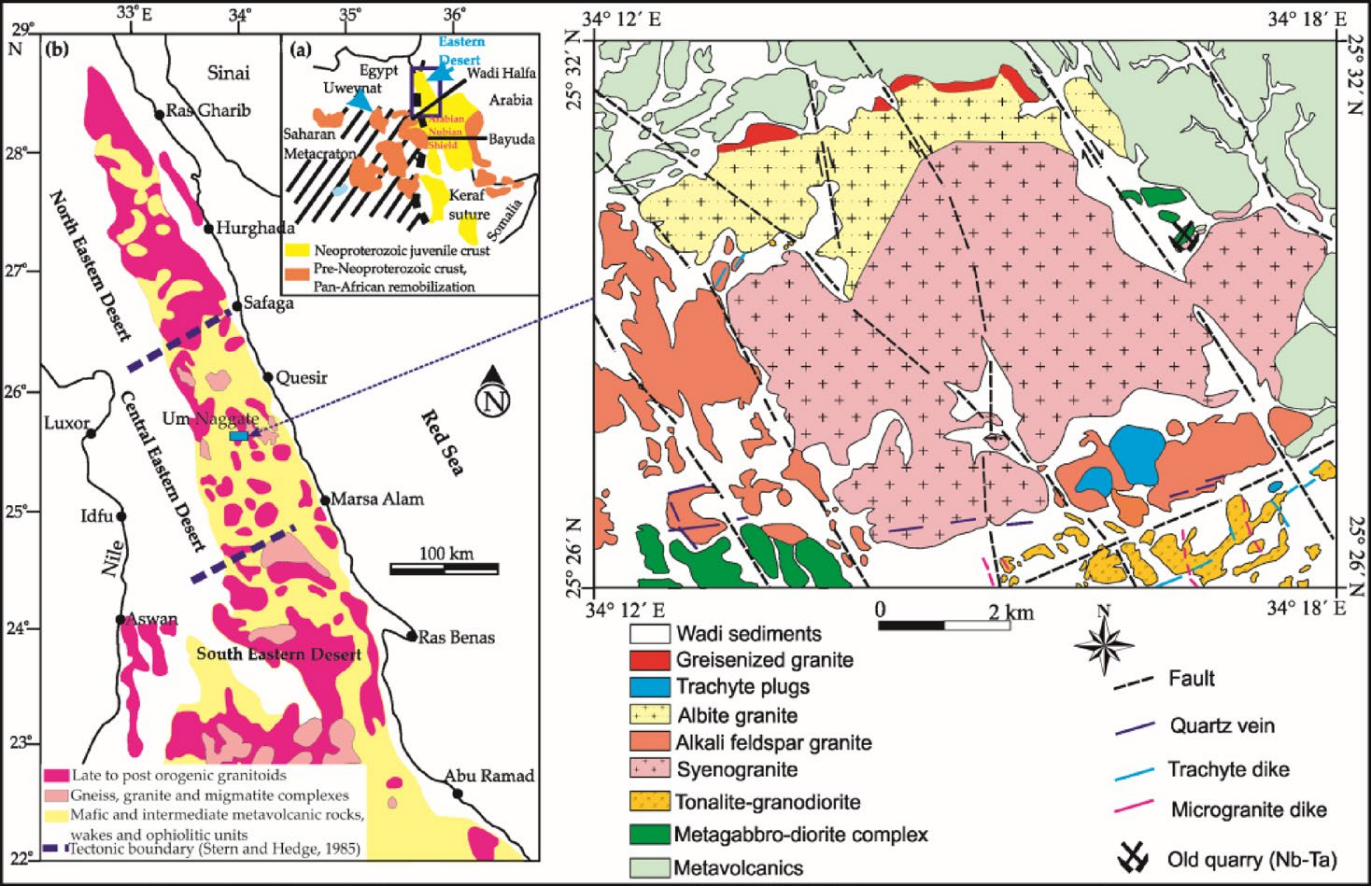
(**a**) Geologic map of the Arabian Nubian Shield (ANS); (**b**) Geologic map showing the Neoproterozoic basement complex in the Eastern Desert (ED) of Egypt^29^; (**c**) Geologic map of Um Naggat, Central Eastern Desert, Egypt. Modified after^23,32,34^. The map is drawn by Coreldraw 12 software for free trial (https://www.coreldraw.com/en/).

Um Naggat area is situated in the Central Eastern Desert of Egypt and forming part of the Arabian-Nubian Shield (ANS)^29^ (Fig. 1a,b). The investigated Um Naggat region consists mainly of the Precambrian basement complex, comprising metavolcanics, metagabbros, tonalite-granodiorite, biotite granite, albite granite and trachyte plugs (Fig. 1c).

Metavolcanics in the investigated Um Naggate area are categorized as older one which have identified as a component of the ophiolite mélange association (El-Gaby et al., 1988; Stern, 1981;^30,31^. They occur as fine-grained, dark gray, red to pale green predominantly in the northern and eastern corners of Um Naggat plutons, and they are distinguished by moderate to high relief. These rocks comprise basic to acidic volcanic varieties including metabasalt, meta-andesite, metadacite and metarhyolite interbeded with their pyroclastics.

Metagabbro-diorites occur as massive rocks, black, green to dark green, exposed locally in the southwestern and central eastern corners of the investigated area. These rocks reveal moderate relief, and have a remarkable sharp intrusive contacts with the exposed granites.

Older granitoids (tonalite-granodiorite) are found as low-relief masses and are distinguished by exfoliation structures. They manifest as fine- to coarse-grained, and vary from whitish gray to pale gray colors. These granitoids were essentially cover the southwestern corners of the investigated area as small scattered plutons. Older granitoids are frequently intruded by other younger granitic rocks (syenogranite and alkali feldspar granite) with sharp intrusive contacts.

The younger granites (syenogranite, alkali feldspar granite and albite granite) plutons represent the most predominated rocks in Um Naggate region. Syenogranite extends in an E–W direction, and reveal the highest relief, oval-shaped, medium to coarse-grained, buff to reddish brown colors. It has irregular boundaries and frequently hosts remarkable xenoliths from the surrounding rocks in the study area^31^.

Alkali feldspar granite occurs medium to coarse-grained, red to pinkish red and buff colors, and showing equigranular to porphyritic textures. It is bounded the syenogranite particularly in the southern and western parts of the investigated area^32^. It consists essentially of K-feldspar, quartz, plagioclase, biotite and muscovite.

**Fig. 2:**
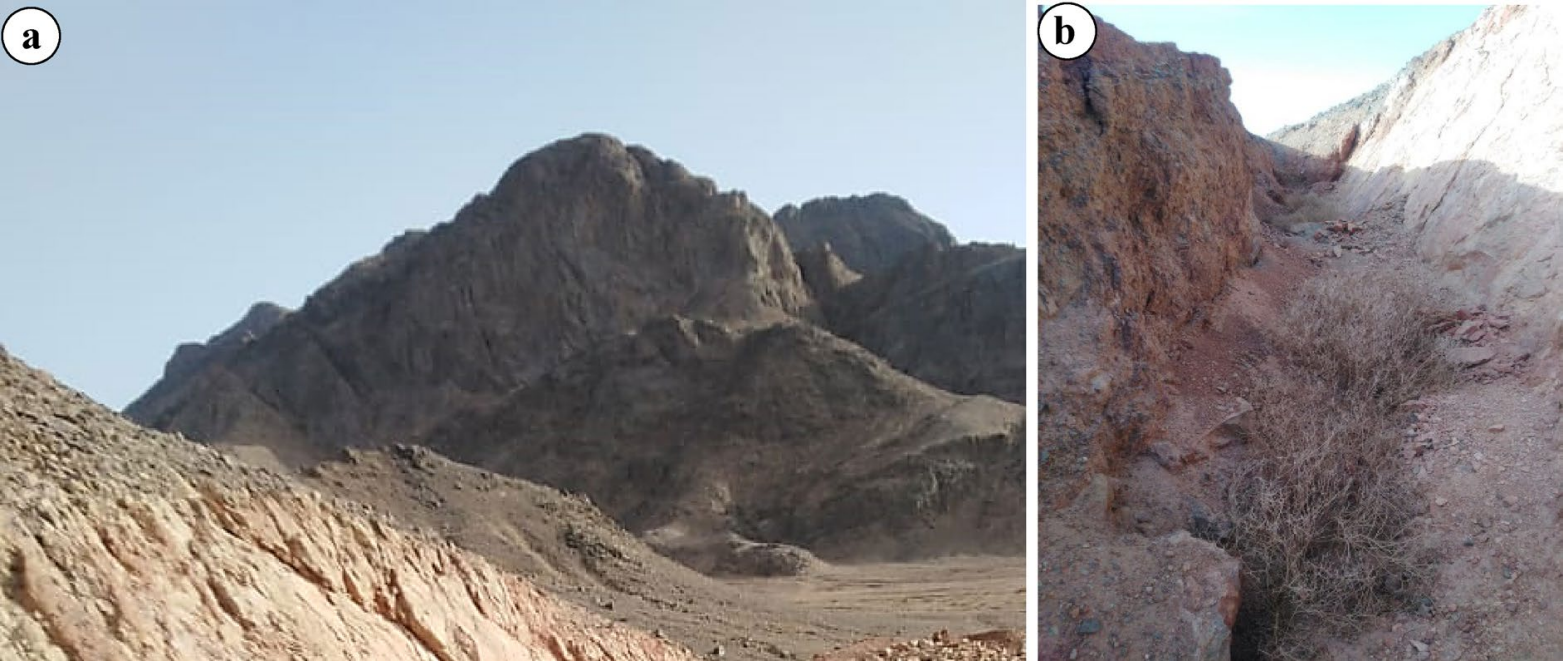
(**a**) High-relief albite granite pluton, (**b**) Trench in mineralized greisen.

The albite granite is well exposed as dome-shaped intrusion with remarkable irregular boundaries in the northwestern side and flatness in the E–W trend (Fig. 2a). It manifest as medium to-coarse-grained, whitish color, and possessing moderate to high relief. Hydrothermal alteration has extensively affected the albite granite including greisenization, kaolinization and fluoritization, coinciding with sinistral fault system striking NW trend (Fig. 2b). This rock has undergone post-magmatic metasomatism, resulting in the formation of rare metals such as Nb, Ta, Zr, F, Th and U, particularly in greisen body^22,31,33^. Columbite and tantalite encountered with minor amounts of cassiterite, zircon and thorite are well recorded^33^. Pegmatite bodies frequently occur as lens-shape at the peripheral margins of the albitized granite. This bodies rich in Nb-Ta bearing mineralizations encountered with garnet and fluorite.

Greisenized granite manifest as fine-grained, highly altered rocks, vary from gray to greenish gray color, and is characterized by the development of an assemblage mainly consisting of muscovite, quartz, sericite and fluorite as well as rare metal mineralization as mentioned in the present study. Greisenization involves feldspar destabilization and H^+^ consumption, resulting in the stable formation of muscovite, quartz, and fluorite. The greisen zones manifest as lenticular shape, vary from 5 to 25 m width, and from 100 to 400 m in their length. It is confined exclusively to E–W, NNE, and NW fractures along the intrusive contact between the metavolcanics and albite granite. In progression from the albitized granite to greisenized granite, the hydrothermal alteration proceeds from selectively pervasive the development of muscovite and sericite on expense of Na- and K-feldspar, which have a great effect on the most original rock-forming minerals.

Consequently, all the original constituents of abitized granite are no longer stable, and are mostly become transformed into the greisen assemblages as resulted in the following reactions of Hemley and Jones^35^ as follows:

1 $$\begin{aligned} & {\mathrm{3KAlS}}{{\mathrm{i}}_{\mathrm{3}}}{{\mathrm{O}}_{\mathrm{8}}}{\mathrm{K}} - {\mathrm{feldspar}}\, + \,{\mathrm{2}}{{\mathrm{H}}^ + }{\text{acidic fluids}} \to {\text{ KA}}{{\mathrm{l}}_{\mathrm{3}}}{\mathrm{S}}{{\mathrm{i}}_{\mathrm{3}}}{{\mathrm{O}}_{{\mathrm{1}}0}}{\left( {{\mathrm{OH}}} \right)_{\mathrm{2}}}\left( {{\text{muscovite or sericite}}} \right) \\ & + {\text{ 2}}{{\mathrm{K}}^ + } + {\text{ 6Si}}{{\mathrm{O}}_{\mathrm{2}}}{\mathrm{quartz}} \end{aligned}$$

2 $$\begin{aligned} & {\mathrm{6NaAlS}}{{\mathrm{i}}_{\mathrm{3}}}{{\mathrm{O}}_{\mathrm{8}}}{\mathrm{Na}} - {\mathrm{plagioclase}}\, + \,{\mathrm{2}}{{\mathrm{K}}^ + } + {\text{ 4}}{{\mathrm{H}}^ + } \to {\text{ 2KA}}{{\mathrm{l}}_{\mathrm{3}}}{\mathrm{S}}{{\mathrm{i}}_{\mathrm{3}}}{{\mathrm{O}}_{{\mathrm{1}}0}}{\left( {{\mathrm{OH}}} \right)_{\mathrm{2}}}\left( {{\text{muscovite or sericite}}} \right) \\ & + {\mathrm{12Si}}{{\mathrm{O}}_{\mathrm{2}}}{\mathrm{quartz}} + {\mathrm{6N}}{{\mathrm{a}}^ + } \end{aligned}$$

During the processes of Na-metasomatism (albitization), the anorthite has been transformed to albite with a marked liberation of calcium^36^ as follows:

3 $${\mathrm{2N}}{{\mathrm{a}}^ + } + {\text{ 4Si}}{{\mathrm{O}}_{\mathrm{2}}}\, + \,{\mathrm{CaA}}{{\mathrm{l}}_{\mathrm{2}}}{\mathrm{S}}{{\mathrm{i}}_{\mathrm{2}}}{{\mathrm{O}}_{{\text{8 anorthite}}}} \to {\mathrm{2NaAlS}}{{\mathrm{i}}_{\mathrm{3}}}{{\mathrm{O}}_{{\mathrm{8}}\;{\mathrm{illite}}}} + {\text{ C}}{{\mathrm{a}}^{{\mathrm{2}} + }}$$

The acidic alteration of Biotite could be able to form illite or sericite and quartz with remarkable liberation of fluorine, magnesium, iron and oxygen in the hydrothermal fluids^37^ as follows.

4 $$\begin{aligned} & {\mathrm{3}}{\left[ {{\mathrm{K}}{{\left( {{\mathrm{Mg}},{\mathrm{Fe}}} \right)}_{\mathrm{3}}}\left( {{\mathrm{AlS}}{{\mathrm{i}}_{\mathrm{3}}}{{\mathrm{O}}_{{\mathrm{1}}0}}} \right){{\left( {{\mathrm{OH}},{\mathrm{F}}} \right)}_{\mathrm{2}}}} \right]_{{\mathrm{biotite}}}} + {\text{ 2}}{{\mathrm{H}}^ + }_{{\text{acidic fluids}}} \to {\mathrm{KA}}{{\mathrm{l}}_{\mathrm{2}}}\left( {{\mathrm{S}}{{\mathrm{i}}_{\mathrm{3}}}{\mathrm{Al}}{{\mathrm{O}}_{{\mathrm{1}}0}}} \right){\left( {{\mathrm{OH}}} \right)_{{\mathrm{2}}\;{\mathrm{illite}}}} \\ & + {\text{ 2K}}\left( {{\mathrm{OH}}} \right)\, + \,{\mathrm{9}}{\left( {{\mathrm{Mg}},{\mathrm{Fe}}} \right)^{{\mathrm{2}} + }} + {\text{ 6Si}}{{\mathrm{O}}_{{\mathrm{2}}\;{\mathrm{quartz}}}} + {\text{ 2}}{{\mathrm{H}}_{\mathrm{2}}}{\mathrm{O}}\, + \,{\mathrm{8}}{{\mathrm{O}}^{{\mathrm{2}} - }} + {\text{ 6}}{{\mathrm{F}}^ - }_{({\mathrm{aq}}.)} \end{aligned}$$

The released 3Ca^2+^ and 6 F^−^ during illitization (reaction 3 and 4) of the anorthite and biotite may combine together forming fluorite 3CaF_2_ which is observed within the greisenized granite.

In places, zone of greisenization grades into fluoritization, which is manifested well by the development of greisenized granite zone rich in flourite.

Trachyte plugs are fine-to medium-grained, red to burned brown and brown colors, and showing medium-releif. They are cut through the tonalite-granodiorite and syenogranite in the E–W and NW directions (Meneisy^45^). Structurally, the granitic rocks were affected by fault systems, dike swarms and quartz and fluorite veins, such as E–W, NW, NE and N–S (Khalifa^46^)^33^. These granitic plutons were affected by different hydrothermal alterations albitization, greisen, kaolinization and fluoritization^23^.

## Materials and methods

Three representative bulk samples (approximately 10 kg each) were collected from spatially separated surface exposures of the greisenized granite bodies in Um Naggat area. Sampling locations were selected based on field geological observations to encompass the range of alteration intensity, fluorite mineralization, and mineralogical characteristics observed within the exposed greisen zones. The collected samples were intended to provide representative material for mineralogical characterization and beneficiation studies rather than for resource estimation.

Representative portions of the samples were prepared as thin sections for petrographic and mineralogical examination under transmitted and reflected light microscopy. Bulk samples were initially crushed using a jaw crusher and subsequently ground in a Denver rod mill to achieve a particle size of less than 2000 μm. Representative subsamples were obtained using a riffle splitter to ensure homogeneity, and these subsamples were then further ground to below 63 μm using a disc mill to obtain material suitable for chemical analysis. Major and trace elements were determined by X-ray fluorescence (XRF) spectroscopy using a Rigaku energy-dispersive X-ray fluorescence (EDXRF) spectrometer equipped with polarized optics. Analyses were performed on 10 g of finely ground sample. The instrument, fitted with a 50 W Pd-target X-ray tube and operated with RPF-SQX software, enabled standardless quantification based on the fundamental parameter method and full-profile spectral fitting. Calibration was achieved through optimized fundamental parameters and Rayleigh scattering corrections. According to the XRF analytical specifications provided by the laboratory, the detection limits were approximately 20 ppm (equivalent to 0.002 wt%) for major-element oxides and approximately 2 ppm for trace elements. Quality control procedures included repeated measurements and internal instrument stability checks to ensure analytical reliability and reproducibility. The remaining ground feed sample (less than 2000 μm) was deslimed using a desliming cone to quantify the fine particle fraction. Grain size distribution was determined using a mechanical sieve shaker fitted with ASTM-standard sieves ranging from 2000 to 63 μm, producing six size fractions: 1000, 750, 500, 250, 125, and 63 μm. Each fraction was weighed and recorded for subsequent mineralogical and beneficiation studies.

Representative 100 g subsamples of the bulk material were subjected to density separation procedures to determine the proportion and characterize the heavy mineral assemblage. The separation was carried out using a separatory funnel. Initially, bromoform (SG 2.89) was added, followed by the sample, and the mixture was thoroughly stirred to ensure complete dispersion. The system was then allowed to settle undisturbed for approximately 5 min to facilitate gravity separation, after which the heavy fraction was collected by opening the funnel tap. The recovered heavy fraction was subsequently washed with acetone, dried, and weighed.

The dried heavy fraction obtained from the bromoform stage was then transferred to a second separatory funnel containing methylene iodide (SG 3.3) for further separation of higher-density mineral phases. The mixture was again thoroughly stirred and allowed to settle for approximately 5 min, after which the denser fraction was collected by opening the funnel tap. Both the heavy and light fractions obtained from the methylene iodide separation were washed with acetone, dried, and weighed to determine their respective proportions relative to the bulk sample. Representative grains of each identified mineral phase were handpicked from the heavy and light fractions under an Olympus stereobinocular microscope based on optical homogeneity criteria, including uniform color, consistent luster, and the absence of visible inclusions or intergrowths. Approximately 20 grains of each mineral phase, where available, were selected for qualitative mineralogical characterization and confirmation by binocular microscopy, XRD, and SEM-EDS/BSE analyses. These grains were not used for quantitative statistical assessment of mineral abundances.The selected grains were examined using a Philips XL 30 scanning electron microscope (SEM) equipped with an energy-dispersive spectrometer (EDS) for compositional analysis. SEM analyses were conducted at an accelerating voltage of 30 kV, with a beam diameter of approximately 1 μm and counting times ranging from 60 to 120 s. The minimum detectable element concentrations ranged from 0.1 to 1 wt%, ensuring reliable semi-quantitative compositional determination.

Prior to beneficiation testing, the entire comminuted bulk sample (~ 20 kg) was thoroughly homogenized to ensure sample representativeness. A representative subsample was then subjected to heavy-liquid separation for determination of the feed THM content. Likewise, the gravity concentrate recovered from the shaking table was homogenized prior to subsampling and heavy-liquid separation analysis. The THM grades obtained from these representative subsamples were subsequently used for material-balance calculations and recovery estimation.

A composite feed sample (~ 20 kg) was prepared by thoroughly homogenizing equal weight proportions of three representative greisenized granite samples. This composite sample was used for beneficiation experiments aimed at the physical separation of fluorite and associated rare-metals mineralization. The feed material was subjected to wet gravity concentration using a laboratory Wilfley shaking table (Model No. 13) to exploit differences in specific gravity between target heavy minerals and the lighter gangue components.

To obtain clean concentrates of total heavy minerals (THMs), wet gravity separation was conducted in two sequential stages (rougher and scavenger) under optimized operating conditions designed to maximize both recovery and grade. The selected operating parameters were established based on preliminary experimental trials, supported by literature data for similar gravity separation systems, and were adjusted according to the particle size distribution, liberation characteristics, and density contrast of the studied material.

In the rougher stage, the feed was processed at a rate of 140 kg/h, with a water flow rate of 15 L/min, a stroke length of 2 mm, and a table inclination of 15°. The resulting tailings were subsequently treated in a scavenger stage to recover residual heavy minerals. This stage was operated at a feed rate of 130 kg/h, with a water flow rate of 10 L/min, a stroke length of 1.5 mm, and a table inclination of 8°. This two-stage approach enabled efficient concentration of the heavy mineral fraction while effectively rejecting silicate gangue minerals, primarily quartz and feldspar.The gravity-concentrated fraction was subsequently processed using a Carpco dry high-intensity magnetic separator (DHIMS; Model MIH III-5) to separate ferro- and paramagnetic minerals from the diamagnetic fraction, thereby enhancing the recovery and purity of the target minerals.

Final concentrates from the gravity and magnetic separation processes were analyzed using SEM-EDS and backscattered electron (BSE) imaging to determine the mineralogical composition and verify the effectiveness of the physical separation procedures.

After each separation stage, representative samples of approximately 100 g were collected for characterization. These samples were examined using scanning electron microscopy (SEM), X-ray diffraction (XRD), and heavy liquid separation to support concentrate characterization and material balance calculations. X-ray diffraction (XRD) analysis was carried out using a Bruker D8 Discover diffractometer equipped with a Cu Kα radiation source (λ = 1.541 Å) operated at 40 kV and 40 mA. Diffraction data were collected in reflection mode over a 2θ range of 5–80° with a step size of 0.05°. Mineral phases were identified by comparison with the ICDD Powder Diffraction File (PDF, 2020 release).

## Results and discussion

###  Petrography of the greisenized granite

**Fig. 3:**
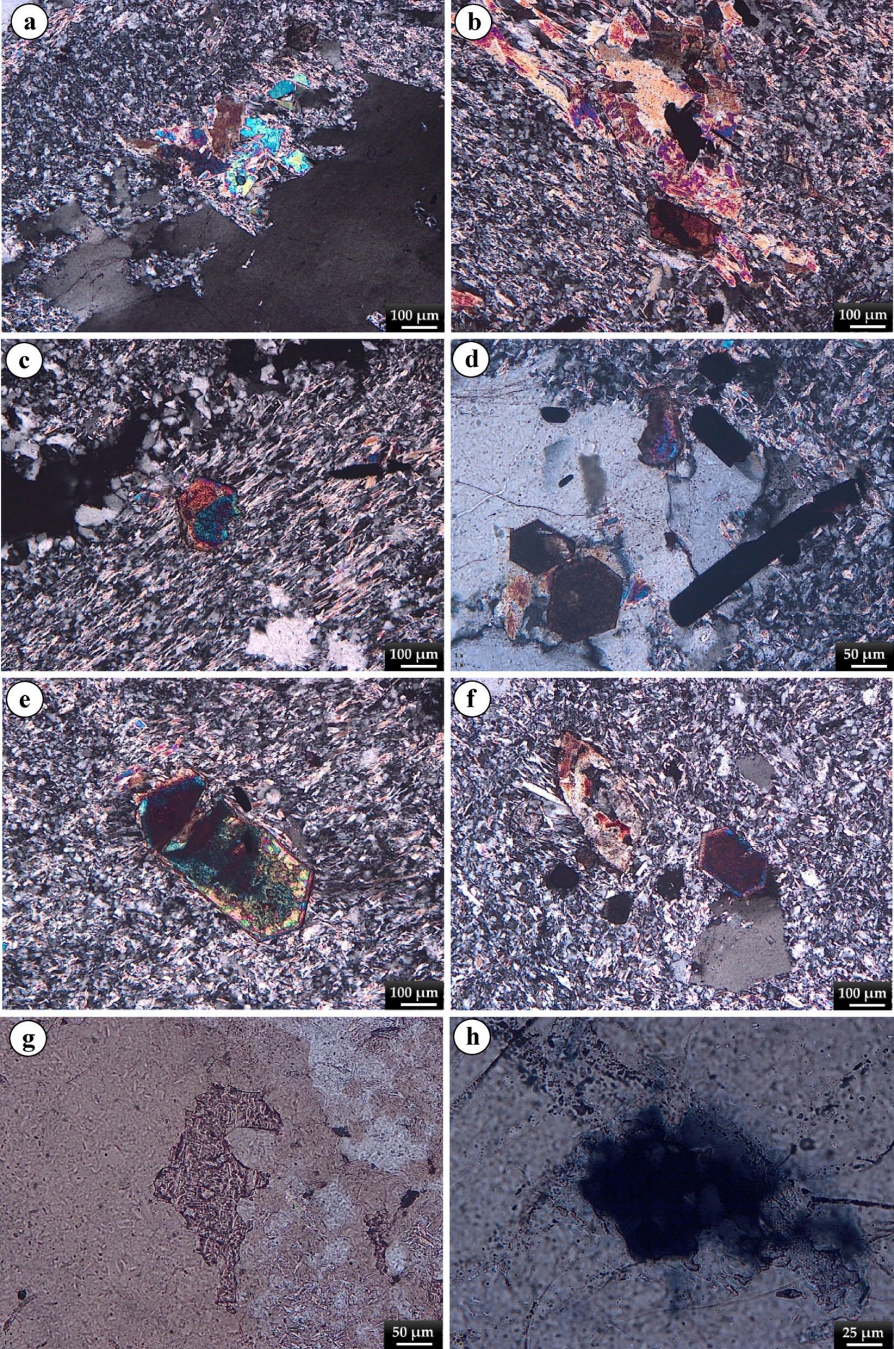
Photomicrographs of the studied greisen at Um Naggate, Central Eastern of Egypt. (**a**) Transformation of minute muscovite into sericite, that encountered with quartz and muscovite; (**b**) Well-formed crystals of titanite associated with muscovite, sericite and opaque minerals; (**c**) Prismatic zircon crystal enclosed in greisen; (**d**) Large quartz crystal encloses zircon and ilmenite which is founded as needle-like appearance; (**e**,**f**) Well-formed zircon crystals enclosed in greisen, (**g**) Anhedral crystal of fluorite is distinguished by its high relief, perfect cleavage and cracks, (**h**) Isotropic fluorite crystal enclosed in quartz. All the photomicrographs have been taken under Crossed Nicols (C.N.), except (**g**) has taken under under polarized light (P.P.L.).

Greisen bodies are well developed exclusively along fractures having E–W, NNE, and NW orientations. The action of emerged greisenizing fluids in the northern corner of the investigated Um Naggat plutons is obviously restricted to subsolidus effects within the albitized granite plutons. While its effects on the surrounding rocks particularly the metavolcanics is completely absent. In progression from albitization into greisen, this style of alteration comprises advanced development of sericite particularly at the expense of Na- and K-feldspar, to pervasive (i.e., all the original rock forming minerals are completely altered and the greisen assemblages are well formed (Fig. 3a–h). However, with increasing the intensity of greisen process, a series of different facies are well developed into five sequences as follow: (a) albitized granite, (b) greisenized granite, (c) muscovite-quartz greisen, (d) muscovite-fluorite-topaz-quartz greisen, and (e) quartz-muscovite greisen core^22^.

The greisen process involves the formation of predominantly fine-grain muscovite, sericite and quartz, as well as minor amounts of accessory minerals including zircon, titanite, fluorite, columbite, tantalite, cassiterite and tourmaline that overprint the albitized granite.

The K-feldspar crystals are completely altered to fine-muscovite flakes and sericite. The interstitial greisen hosts muscovite, quartz, sericite and fluorite (Fig. 3a–h). Biotite flacks are observed, whereas the muscovite crystals are mostly very common, and is often encountered with titanite and zircon crystals. Quartz is found as fine- to coarse-grained, subhedral to anhedral crystals. Some crystals are slightly cracked due to deformation and showing faint to conspicuous undulose extinction. Some crystals appear to be developed due to enlargement of the early-formed quartz (indicating a process of silicification). Muscovite occurs as medium to coarse elongated irregular flakes (Fig. 3a, b). The presence of conspicuous amounts of primary muscovite suggests genesis from peraluminous magma. Most fine- muscovite and sericite appear as an alteration products of biotite and feldspar filling the interstatial spaces between minerals (Fig. 3a–h).

Zircon occurs as prismatic crystals enclosed in quartz and some crystals are present in the alteration products of greisen (Fig. 3c–f). Zircon crystals are usually cloudy and rimmed with iron oxides. Some crystals are partially to completely metamict. Titanite manifests as euhedral to subhedral sphenoid or rhombic crystals. It is encountered with muscovite and alteration products of greisen (Fig. 3b).

Fluorite is usually found as anhedral, cracked, fractured and characterized by its high relief. It reveals perfect cleavage, colorless, faint violet and pale green to pale pink under polarized light and isotropic under crossed nicols (Fig. 3g, h). It is usually enclosed in quartz and/or encountered filling spaces between fine constituents of greisen.

### Sample preparation

**Fig. 4:**
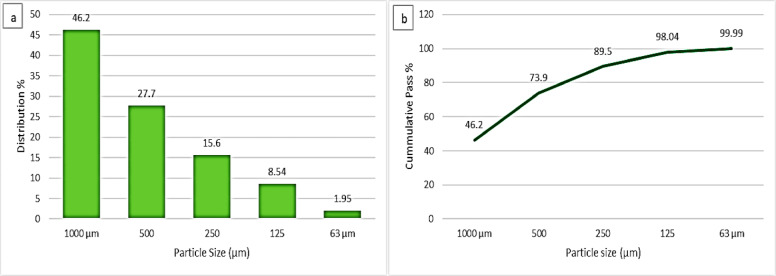
(**a**) Particle size distribution histogram and (**b**) cumulative passing curve of greisenized granite samples from Um Naggat area after comminution.

The particle size distribution analyses of greisenized granite samples from Um Naggat area after comminution are shown in Fig. 4a, b. The results reveal a predominance of finer fractions, with 46.2% of the material retained at 1000 μm, 27.7% at 500 μm, 15.6% at 250 μm, 8.54% at 125 μm, and 1.95% at 63 μm. The cumulative passing curve indicates progressive size reduction, with 73.9% passing below 500 μm, 89.5% below 250 μm, 98.04% below 125 μm, and 99.99% below 63 μm.

These data confirm the efficiency of the comminution process, achieving over 98% of the material within the size range of − 2000 + 63 μm, which is considered optimal for achieving sufficient mineral liberation required for subsequent beneficiation by gravity separation (shaking table) and high-intensity magnetic separation. Such effective size reduction enhances mineral liberation, improves the efficiency of physical separation techniques, and maximizes the potential recovery of valuable mineral phases.

Based on density separation using bromoform (SG 2.89) and methylene iodide (SG 3.3), combined with mineralogical investigation under a stereo binocular microscope, it was determined that the bulk sample from Um Naggat area is dominated by light silicate minerals, primarily quartz with minor feldspar, comprising approximately 98.05% of the total sample. This high proportion of light silicates is consistent with the greisenization of Um Naggat granite.

The total heavy mineral fraction constitutes approximately 1.95 wt% of the bulk sample, as determined from the weight of the bromoform heavy fraction (specific gravity > 2.89). Subsequent separation using methylene iodide (SG 3.3) allowed further subdivision of this fraction. The fluorite-rich fraction accounts for approximately 1.47 wt% of the bulk sample, corresponding to the methylene iodide light fraction (specific gravity between 2.89 and 3.3). The remaining 0.48 wt% of the bulk sample represents the methylene iodide heavy fraction (specific gravity > 3.3), which comprises associated heavy minerals, including zircon, columbite, ilmenite, cassiterite, uranothorite, monazite, aegirine, almandine garnet, iron oxides, and mica. These findings indicate that, although the overall abundance of heavy minerals in the bulk sample is relatively low, the fraction is significantly enriched in valuable phases, particularly fluorite and associated rare-metals minerals. This enrichment, combined with the demonstrated efficiency of the applied beneficiation techniques, highlights the potential for selective recovery and upgrading of economically important mineral phases from the greisenized granite.

### Mineralogical and chemical characterization

**Fig. 5:**
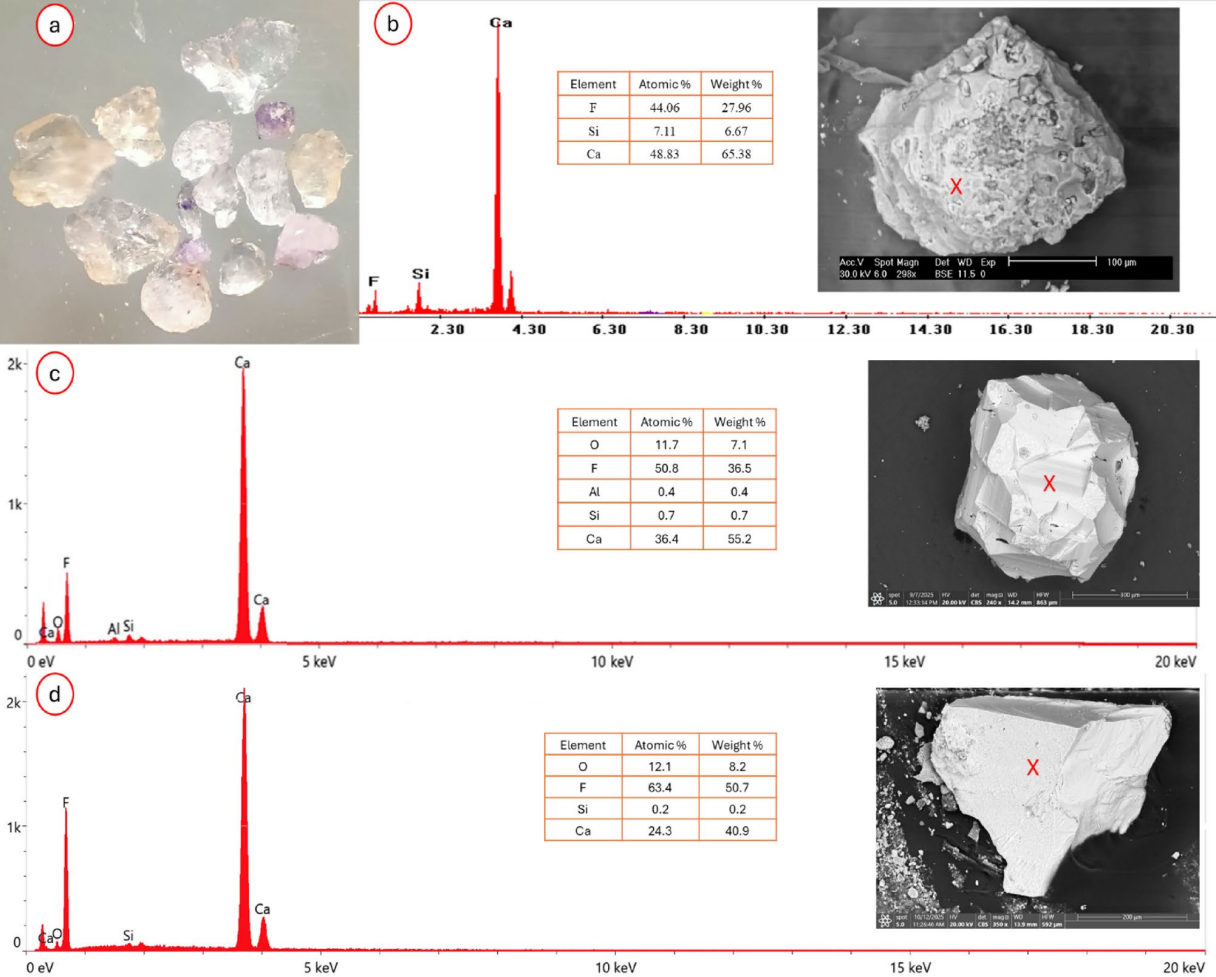
(**a**) stereoscopic image of fluorite grains separated from Um Naggat area, Central Eastern Desert, Egypt, and (**b**–**d**) SEM-BSE/EDS images and spectra illustrating the crystal morphology and chemical composition of several representative fluorite grains.

Detailed mineralogical characterization of the separated minerals was conducted to determine their composition, morphology, and textural features using stereomicroscopic examination and SEM-EDS/BSE analyses. The identified minerals are documented as follows:

*Fluorite* grains separated from Um Naggat area exhibit a range of colors and morphologies, varying from colorless to pale violet (Fig. 5a). Such color variations are commonly attributed in the literature to differences in trace-element composition and crystal growth conditions^38^. These grains are distributed across all size fractions, from coarse particles exceeding 1 mm to fine fractions down to approximately 0.075 mm, indicating a broad size variability within the sampled material. SEM-EDS and BSE analyses conducted on fluorite grains of different colors indicate that fluorite is the predominant mineral phase (Fig. 5b–d), characterized by strong calcium and fluorine signals and only minor detectable silicate and aluminosilicate impurities. The crystals are mainly euhedral to subhedral, with smooth external surfaces and relatively homogeneous internal textures. Inclusions are scarce, and no significant intergrowth with gangue minerals was observed, suggesting favorable liberation characteristics for physical beneficiation. The observed mineralogical characteristics suggest that the fluorite may be amenable to further upgrading through beneficiation; however, detailed chemical analyses are required to assess compliance with specific industrial-grade specifications, including acid-grade fluorite requirements (> 97% CaF₂) that used in hydrofluoric acid production, as well as ceramic- and metallurgical-grade applications (Catuneanu^47^)^39^.

**Fig. 6:**
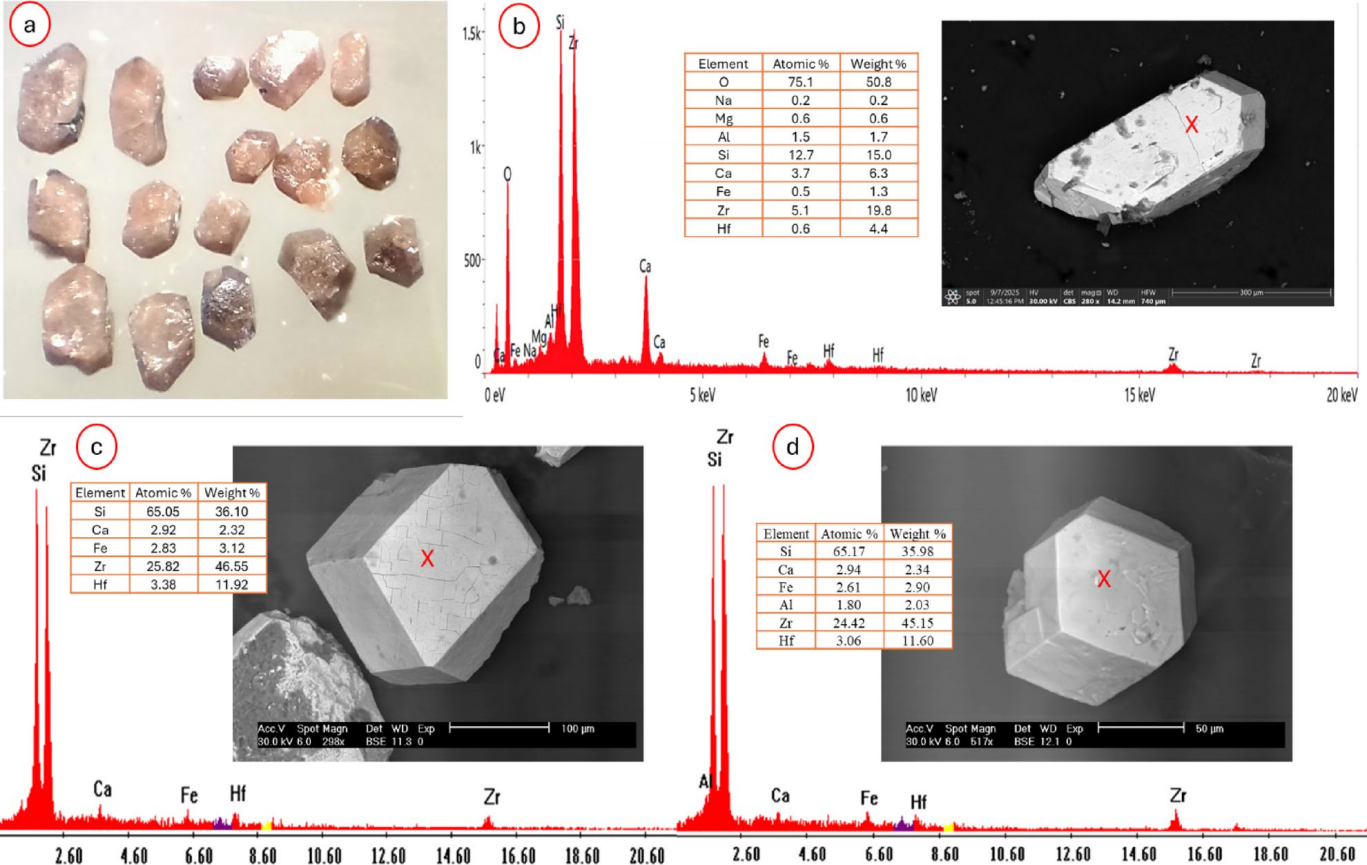
(**a**) stereoscopic image of zircon grains separated from Um Naggat area, Central Eastern Desert, Egypt, and (**b**–**d**) SEM-BSE/EDS images and spectra illustrating the crystal morphology and chemical composition of representative zircon grains.

*Zircon* grains separated from Um Naggat area occur across all size fractions, similar to the fluorite grains, ranging from coarse (> 1 mm) to fine (< 0.075 mm) particles. Under the stereomicroscope, they exhibit well-formed euhedral to subhedral prismatic and bipyramidal crystals with colors varying from light to reddish brown (Fig. 6a). SEM-BSE imaging highlights their distinct crystal habits, smooth and highly reflective surfaces, and the general absence of inclusions or surface alteration features, indicating good preservation and compositional purity (Fig. 6b-d). SEM-EDS analyses confirm a typical zircon composition dominated by strong Zr and Si peaks, consistent with near-stoichiometric ZrSiO₄. Minor elements such as Hf, Fe, Ca, Al, Na, Mg, and trace amounts of U and Th were also detected. Hf is commonly incorporated into the zircon lattice through substitution for Zr, while U and Th are also well-known to occur as lattice-bound elements. In contrast, the presence of Fe, Ca, Al, Na, and Mg is less typical of primary zircon composition and is more likely related to submicroscopic inclusions, surface contamination, or secondary alteration processes, rather than true structural substitution.

**Fig. 7:**
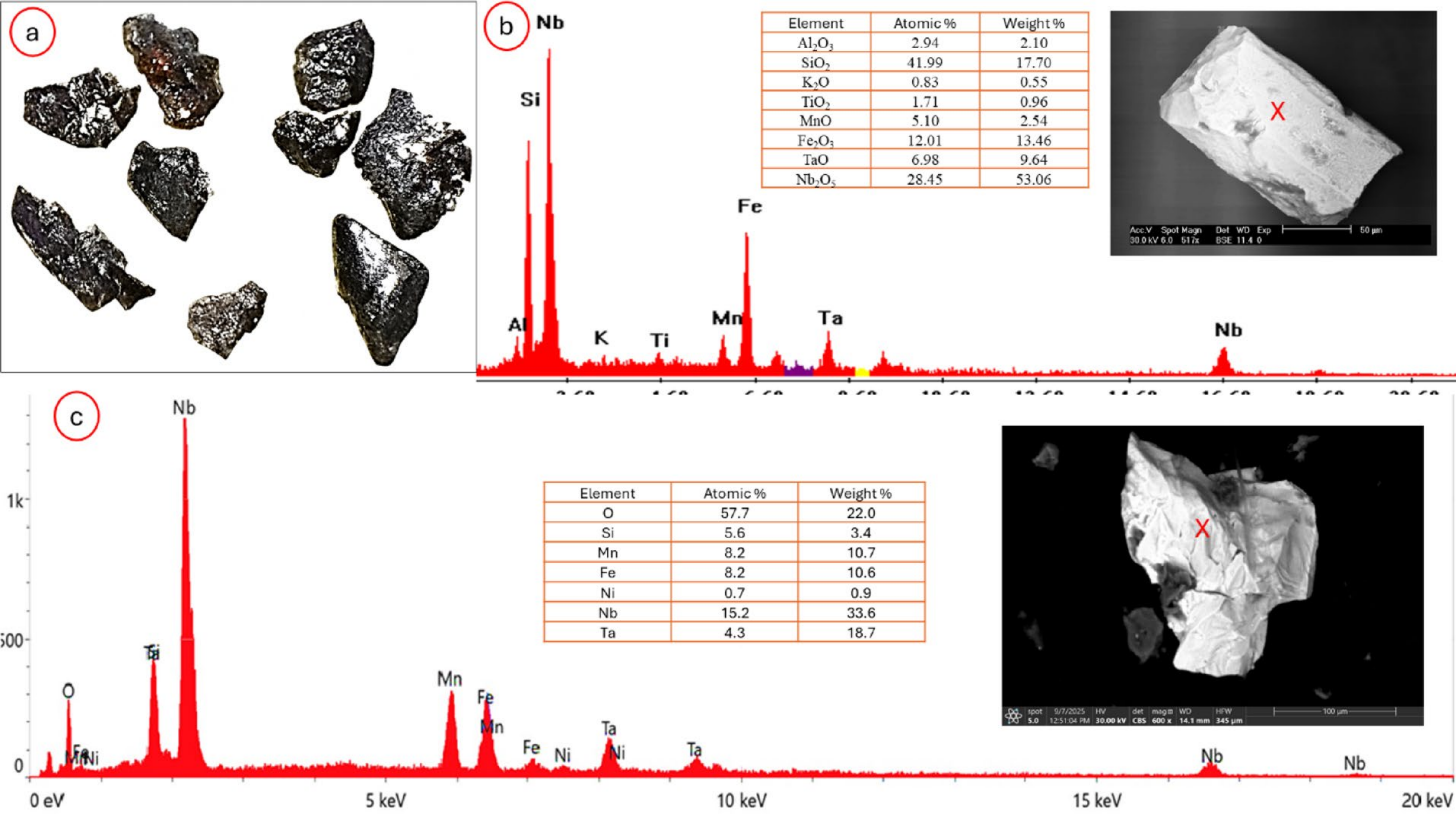
(**a**) Stereoscopic image showing columbite grains separated from Um Naggat area, Central Eastern Desert, Egypt; (**b** and **c**) SEM-BSE/EDS image and spectrum of columbite grains, illustrating their crystal morphology and representative chemical composition.

*Columbite* grains (ferrocolumbite and manganocolumbite) from Um Naggat area are predominantly concentrated in the fine size fractions, generally below 0.25 mm. Under the stereomicroscope, they exhibit dark brown to black coloration and irregular to subhedral morphologies (Fig. 7a). SEM-BSE imaging reveals angular crystal outlines with moderate surface roughness and occasional internal fractures, features indicative of brittle mechanical behavior and minor post-crystallization deformation. Energy-dispersive X-ray spectroscopy (EDS) analyses confirm that the grains are chiefly composed of niobium (Nb) with variable proportions of iron (Fe) and manganese (Mn), accompanied by distinct signals for tantalum (Ta) and titanium (Ti), and minor amounts of Ca, Ni, Al, and other trace elements. SEM-EDS analyses indicate that the analyzed columbite grains exhibit variable Fe/Mn ratios, ranging from approximately 1.0 to 5.3. The grain shown in Fig. 7b is Fe-rich (Fe/Mn ≈ 5.3), corresponding to ferrocolumbite, whereas the grain shown in Fig. 7c exhibits nearly equal Fe and Mn contents (Fe/Mn ≈ 1.0), reflecting compositional variation toward manganocolumbite. These data confirm the predominance of ferrocolumbite while indicating limited substitution toward the manganocolumbite end-member.The SEM–EDS analyses presented in Fig. 7b and c reveal measurable concentrations of Ta and Ti metals in the columbite grains, indicating isomorphic substitution within the columbite–tantalite solid-solution series.

**Fig. 8:**
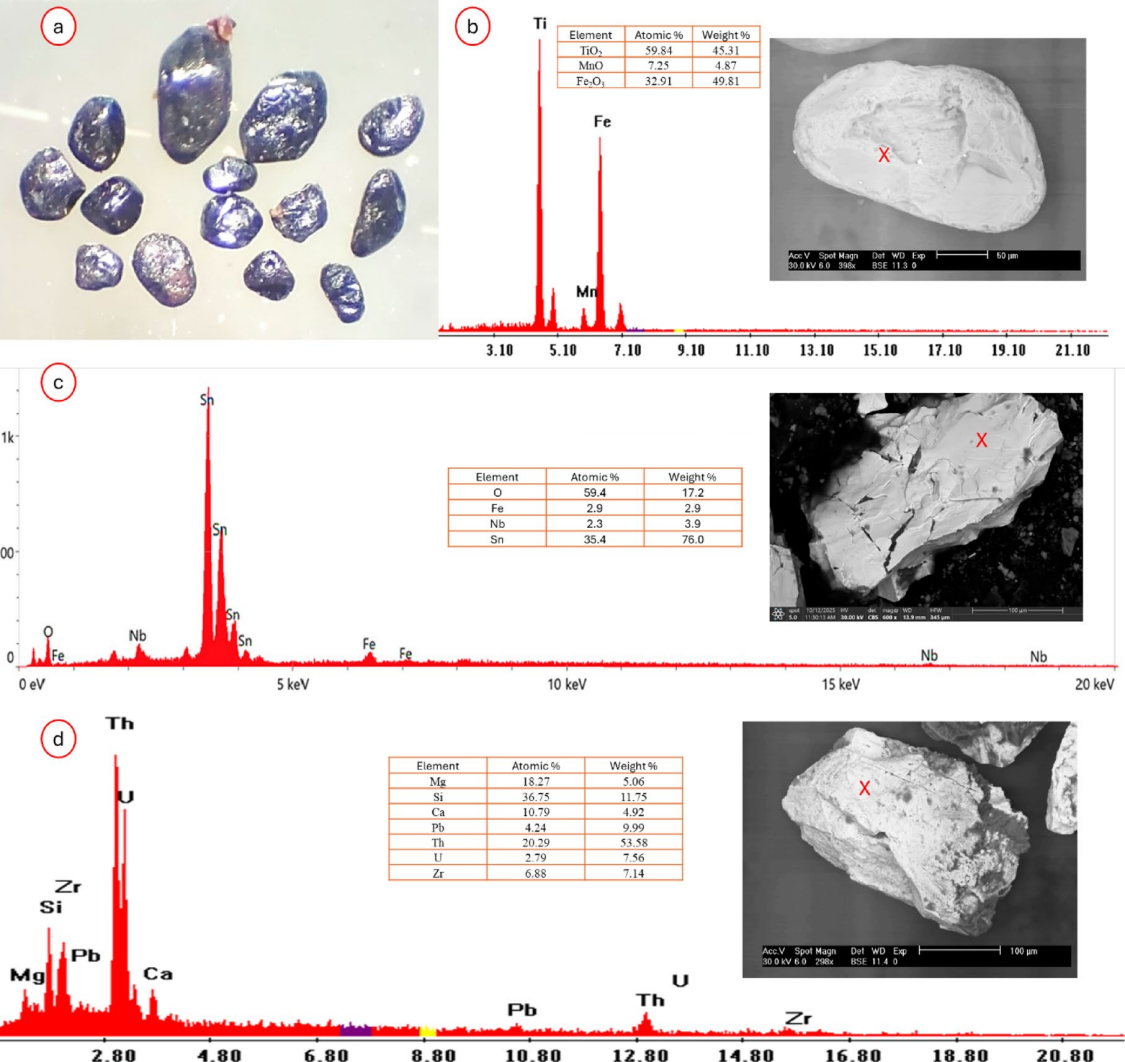
(**a**) Stereoscopic image of ilmenite grains from Um Naggat area, Central Eastern Desert, Egypt; (**b**–**d**) SEM-BSE/EDS images and spectra of ilmenite (**b**), cassiterite (**c**), and uranothorite (**d**), showing crystal morphology and chemical composition.

*Ilmenite*,* cassiterite*,* and uranothorite* occur as trace accessory minerals within the fine-grained fraction (< 0.25 mm) separated from Um Naggat area. Under stereoscopic and SEM-BSE/EDS examination, these phases display distinct morphological and compositional features. Ilmenite grains exhibit a characteristic black metallic luster (Fig. 8a) and are confirmed by prominent Fe and Ti peaks in EDS spectra, consistent with a FeTiO₃ composition and occasional minor Mn or Mg substitution (Fig. 8b). Cassiterite occurs as subrounded to angular grains with a bright, metallic appearance, showing dominant Sn peaks accompanied by minor Fe, Nb, and trace Ta, reflecting typical substitutional trends in granitic and pegmatitic systems (Fig. 8c). Uranothorite is recognized by its light yellow to brown coloration and high backscattered electron intensity; EDS analyses reveal strong Th and U peaks, together with Si and variable Pb and Zr contents, characteristic of thorium–uranium silicate phases (Fig. 8d).

**Fig. 9:**
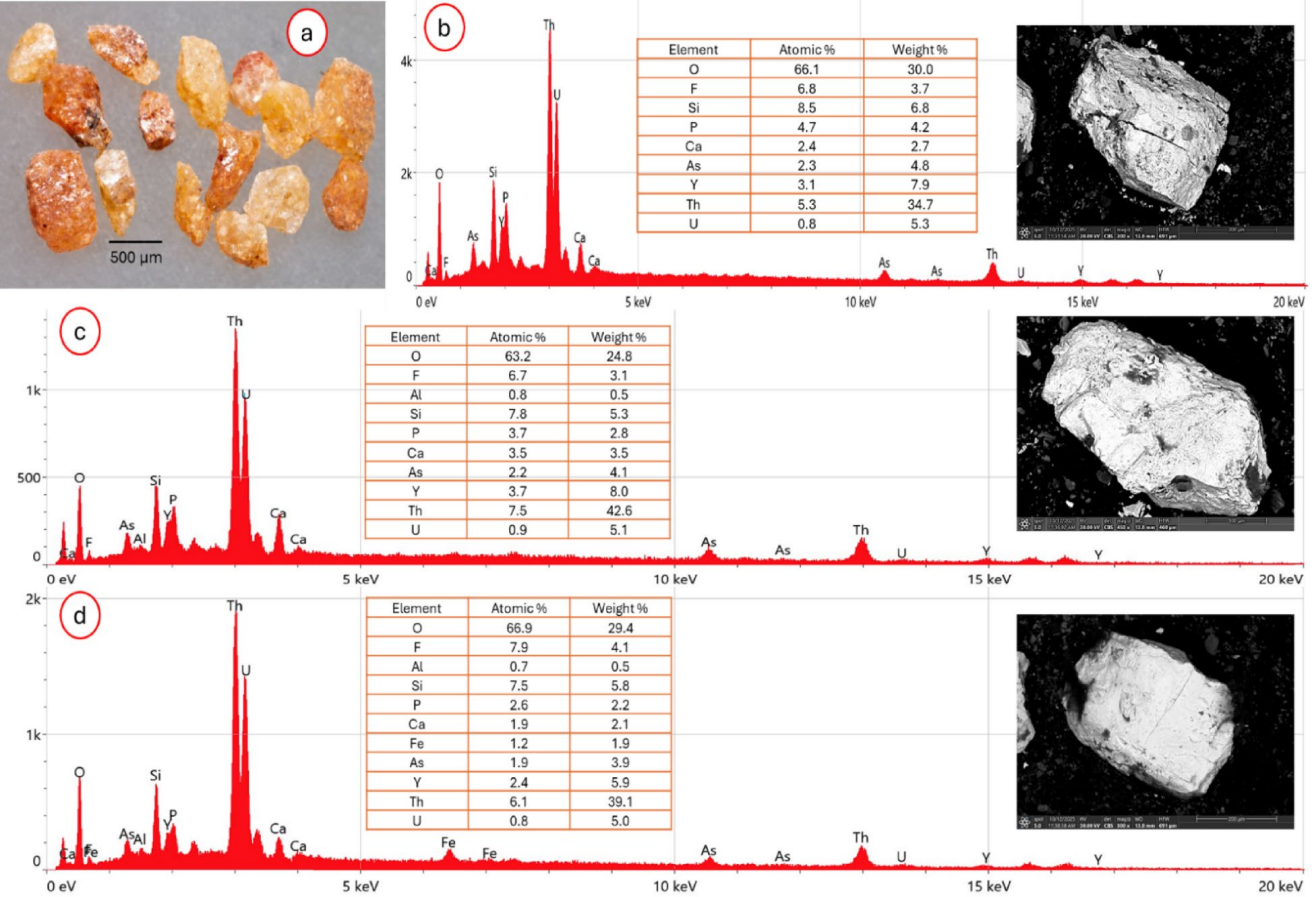
(**a**) stereoscopic image of arsenian thorite crystals separated from Um Naggat area, Central Eastern Desert, Egypt, and (**b**–**d**) SEM-BSE/EDS images and spectra of selected grains.

*Arsenic-bearing thorite* from Um Naggat area (Fig. 9a) occurs as orange-yellow prismatic crystals exhibiting the typical high-luster appearance of Th-silicate minerals enriched in high-field-strength elements. The SEM–BSE images and EDS spectra (Fig. 9b–d) reveal a heterogeneous internal texture, where brighter BSE domains correspond to relatively Th–U-enriched areas. The EDS data consistently indicate dominant Th, U, Si, and O, confirming a thorite-type silicate composition, while subordinate peaks of As, Fe, Ca, Al, and P reflect trace-element associations within the analyzed grains. Arsenic is consistently detected as a minor but persistent element in all analyzed spots. However, EDS analysis provides only semi-quantitative bulk compositional information at the microscale and does not allow discrimination between structural incorporation and the presence of sub-microscopic arsenic-bearing inclusions or surface-related phases. Therefore, the exact mode of occurrence of arsenic cannot be determined from the present dataset.The associated elements (P, Fe, and Al) likely reflect complex trace-element associations typical of Th-rich silicate systems and/or contributions from fine-grained accessory phases. Comparable occurrences of arsenic-bearing thorium silicate assemblages have been reported in rare-metals granite and greisen-related environments, where arsenic is known to occur as a minor element within late-stage mineralization (e.g.^40^).

**Fig. 10:**
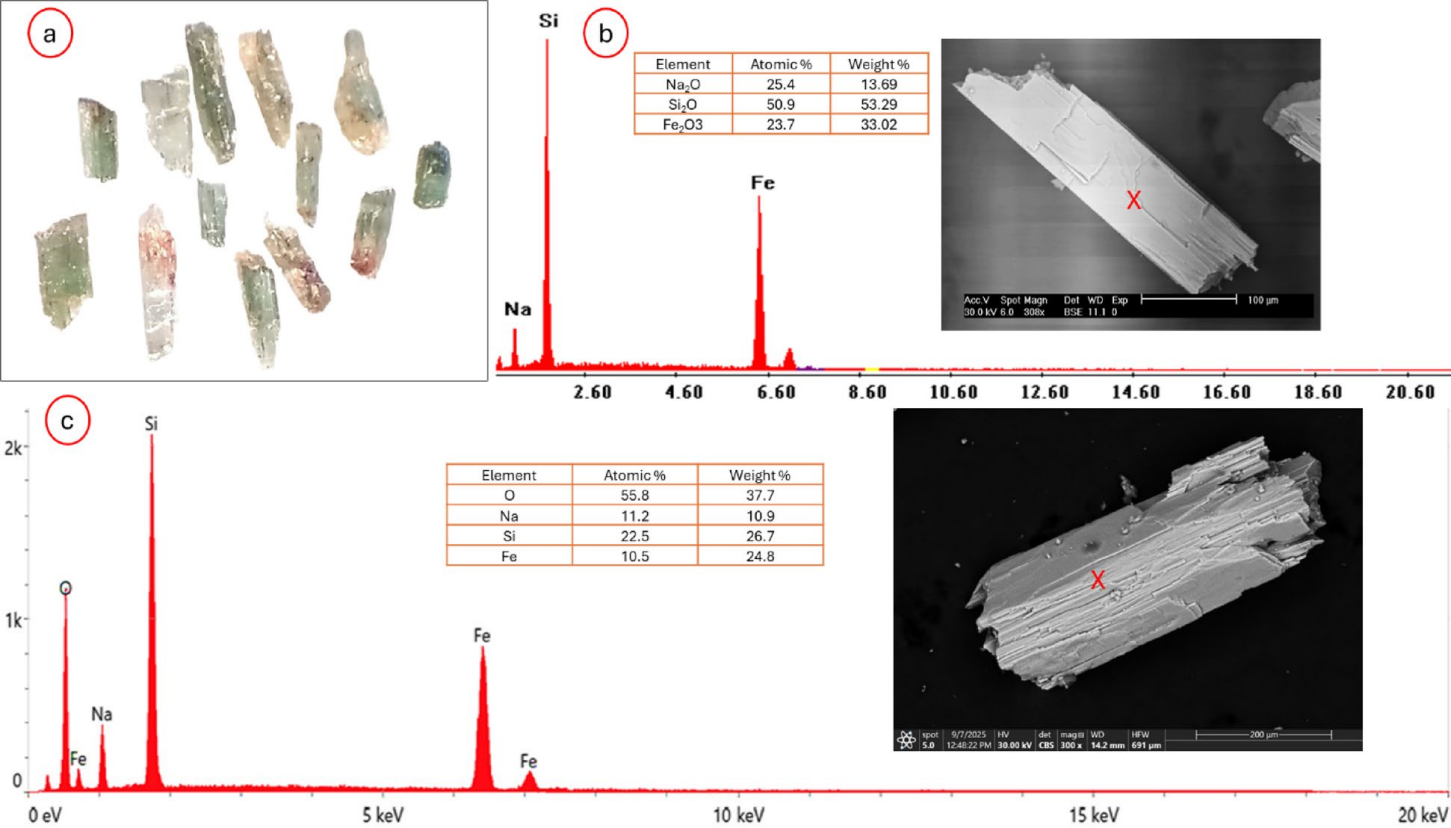
(**a**) stereoscopic image of aegirine (pyroxene group) crystals separated from Um Naggat area, Central Eastern Desert, Egypt, and (**b**,**c**) SEM-BSE/EDS images and spectra depicting their elongated prismatic habit and confirming aegirine composition.

*Aegirine*,* a pyroxene-group mineral*, occurs in all size fractions from coarse (-1 mm) to fine (-0.075 mm) within Um Naggat area. The stereoscopic image (Fig. 10a) shows aegirine grains as elongated, prismatic crystals with colors ranging from pale green to brown. SEM-BSE images (Fig. 10b, c) highlight their elongate habit and well-developed crystal faces, exhibiting a fibrous to columnar texture.

EDS spectra are dominated by strong Si and Na peaks, with prominent Fe signals, consistent with the characteristic composition of aegirine (NaFeSi₂O₆). Minor Mg and occasional Ca are also detected, indicating limited chemical variability within the analyzed grains. The overall chemical composition, characterized by dominant Na, Fe, and Si, supports the identification of the studied phase as aegirine, rather than other clinopyroxene group minerals. These features indicate well-crystallized aegirine and are broadly consistent with its occurrence in evolved alkaline to peralkaline granite systems.

**Fig. 11:**
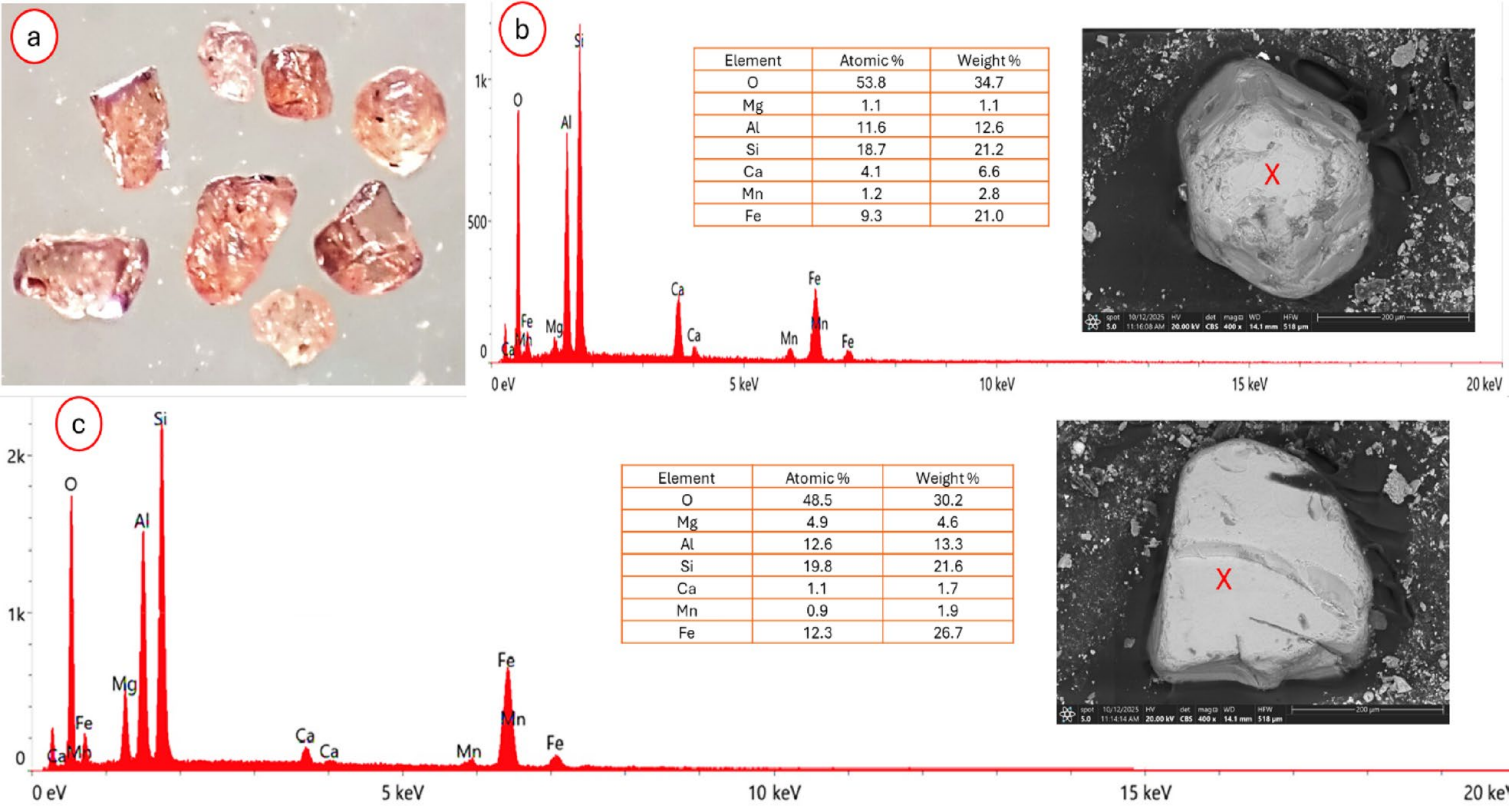
(**a**) stereoscopic image of almandine garnet grains separated from Um Naggat area, Central Eastern Desert, Egypt, and (**b**,**c**) SEM-BSE/EDS images and spectra illustrating their characteristic morphology and chemical composition.

*Almandine garnet* occurs in the coarse fraction, ranging from − 1 mm to + 0.25 mm, within Um Naggat area. In the stereoscopic image (Fig. 11a), the grains are rounded to sub-angular with characteristic reddish-brown hues, reflecting their high iron content. SEM-BSE images (Fig. 11b, c) display well-developed crystal forms with smooth surfaces and occasional minor surface features. EDS spectra reveal dominant Si, Al, and Fe peaks, consistent with almandine composition (Fe₃Al₂(SiO₄)₃), while minor Mg, Ca, and Mn are also detected, representing common lattice substitutions in natural garnets. The combination of high Fe and Al contents, along with the characteristic morphology and coloration, confirms their identification as almandine.

**Fig. 12:**
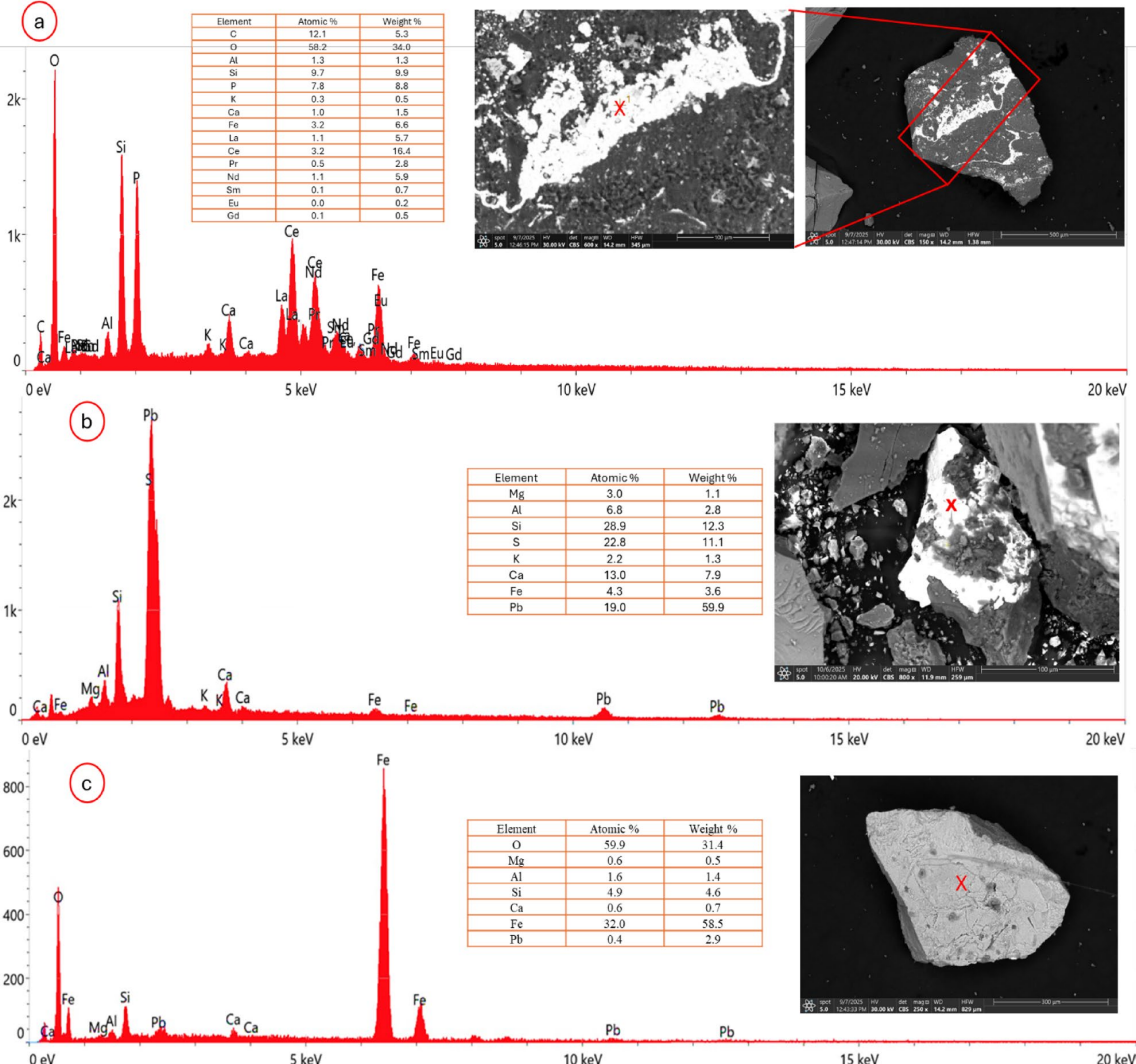
(**a**) SEM-BSE/EDS image and spectrum of a monazite inclusion in a silicate host from Um Naggat area. (**b**) SEM-BSE/EDS image and spectrum of galena inclusions. (**c**) SEM-BSE/EDS image and spectrum of iron oxides.

SEM-BSE imaging of samples from Um Naggat area shows monazite grains hosted within a silicate matrix (Fig. 12a). EDS analysis indicates that these grains are rich in rare earth elements, primarily Ce, La, Nd, Pr, Sm, Gd, and Eu, with minor Ca, Th, and Pb reflecting lattice substitutions and radiogenic effects. The presence of galena (PbS) as inclusions is also confirmed by prominent Pb and S peaks in the SEM-EDS spectrum (Fig. 12b). Associated iron oxides, including magnetite and hematite, occur as granular or vein-like inclusions within the silicate host (Fig. 12c). These observations confirm the coexistence of REE-bearing monazite, galena, and iron oxides in the greisenized granite system.

According to the chemical analyses (Table 1), Um Naggat greisenized granite exhibits a distinctly evolved and silica-rich composition, characterized by a very high SiO₂ content (average = 90.49 wt%), accompanied by moderate Al₂O₃ (1.91 wt%), CaO (1.84 wt%), K₂O (1.68 wt%), and Fe₂O₃ (0.39 wt%). The major oxide total (< 100 wt%) reflects analytical limitations associated with the determination of light elements by the applied XRF method. Consequently, Na₂O and fluorine were not quantitatively reported. Given the abundance of fluorite and the occurrence of Na-bearing minerals identified by mineralogical investigations, the deficit in the oxide total is attributed primarily to the exclusion of fluorine, sodium, and other volatile or non-oxide constituents from the reported analytical dataset. This composition reflects a dominant silicate mineral matrix primarily composed of quartz and feldspars. The relatively low but significant Fe and Al contents further support the presence of ferromagnesian and aluminosilicate phases, notably aegirine (Fe–Na pyroxene) and almandine garnet (Fe–Al garnet), which are consistent with the observed mineral assemblage.

The trace element data (Table 1) highlight remarkable enrichment in several high-field-strength and rare-metal elements. Elevated Nb (960 ppm) and Ta (142 ppm) concentrations indicate substantial columbite-group mineralization, while Zr ranges from 288 to 581 ppm, Hf from 64 to 129 ppm. The Hf/Zr ratio is ~ 0.22, which is extremely higher than typical igneous zircon has Hf/Zr (~ 0.02–0.05). The higher Hf/Zr could be attributed to the enrichment of fluorine (F) as a complexing agent, which facilitate the higher concentration of high field strength elements (HFSE) such as Hf, promoting a remarkable overgrowth of Hf-rich zircon in the hydrothermal fluids^41,42^. Our studies are similar to greisen enriched in Sn, Mo, Be and W deposits are accompanied with granites enriched in Hf relative to Zr and the Hf/Zr ratio < 0.5, while granites enriched in Ta deposits have Hf/Zr < 0.2^43^.

The significant Sn content (327 ppm) corroborates the presence of cassiterite, a key tin-bearing mineral within the greisenized granite. Similarly, elevated U (117 ppm) and Th (187 ppm) point to uranothorite and minor monazite occurrences, consistent with the mineralogical evidence. Enrichment in Y (169 ppm) further suggests the development of complex accessory assemblages such as zircon, monazite, and possibly Y-bearing silicates.

Additionally, the granite shows notable enrichment in Pb (289 ppm) and S (130 ppm), confirming the presence of galena (PbS), as verified by SEM–EDS analyses. The moderate Ti (106 ppm) and Fe contents also support the occurrence of ilmenite. Meanwhile, elevated Rb (429 ppm) and P (345 ppm) values are indicative of alkali feldspar and phosphate mineral phases, respectively. Minor but meaningful concentrations of Mn (146 ppm), V (38 ppm), Zn (53 ppm), and As (40 ppm) reflect the existence of subordinate oxide, sulfide, and arsenate minerals.

**Table 1:** Major and trace element concentrations of Um Naggat greisenized granite determined by XRF analyses.

Elemental analyses	NI	N2	N3	Average
	Major oxides (%)			
SiO_2_	92.83	89.24	89.41	90.49
Al_2_O_3_	1.88	1.97	1.88	1.91
CaO	1.86	1.8	1.86	1.84
K_2_O	1.66	1.7	1.69	1.68
Fe_2_O_3_	0.39	0.39	0.38	0.39
	Trace elements (ppm)			
Nb	937	1007	937	960
Zr	500	581	288	456
P	353	344	339	345
Rb	432	426	429	429
Sn	302	321	358	327
S	104	143	143	130
Pb	288	287	293	289
Th	183	192	187	187
Y	179	166	162	169
Ta	ULD	159	124	142
Mn	146	146	146	146
Ti	108	108	101	106
U	117	125	108	117
Hf	111	129	64	101
V	41	41	32	38
Zn	42	63	55	53
As	ULD	43	36	40

ULD: Under limit of detection.

### Physical separation

#### Gravity concentration

The preceding mineralogical characterization revealed that Um Naggat greisenized granite feed is predominantly composed of light silicate minerals, mainly quartz with minor feldspar, which together constitute approximately 98 wt% of the sample. The targeted heavy minerals, including fluorite, columbite, zircon, cassiterite, and dense silicates such as pyroxene and garnet, account for roughly 1.95 wt% of the feed.

Table 2 presents significant differences in specific gravity between the light and heavy mineral fractions, indicating that effective separation can be achieved using conventional wet gravity techniques, such as shaking tables. Fluorite also demonstrates distinct magnetic behavior compared to most associated heavy minerals, which enables its selective recovery through High-Intensity Magnetic Separation (HIMS). The specific gravities and magnetic properties of fluorite and the accompanying minerals are summarized in Table 2, highlighting the key parameters that govern efficient physical separation.

To assess the suitability of gravity concentration, the concentration criterion (CC) was employed, which is defined as:

$$\:\mathrm{C}\mathrm{o}\mathrm{n}\mathrm{c}\mathrm{e}\mathrm{n}\mathrm{t}\mathrm{r}\mathrm{a}\mathrm{t}\mathrm{i}\mathrm{o}\mathrm{n}\:\mathrm{C}\mathrm{r}\mathrm{i}\mathrm{t}\mathrm{e}\mathrm{r}\mathrm{i}\mathrm{o}\mathrm{n}\:\left(\mathrm{C}\mathrm{C}\right)\:=\frac{\mathrm{S}\mathrm{p}.\:\mathrm{g}\mathrm{r}.\:\mathrm{o}\mathrm{f}\:\mathrm{h}\mathrm{e}\mathrm{a}\mathrm{v}\mathrm{y}\:\mathrm{m}\mathrm{i}\mathrm{n}\mathrm{e}\mathrm{r}\mathrm{a}\mathrm{l}\:-\:\mathrm{S}\mathrm{p}.\:\mathrm{g}\mathrm{r}.\:\mathrm{o}\mathrm{f}\:\mathrm{f}\mathrm{l}\mathrm{u}\mathrm{i}\mathrm{d}\:\mathrm{m}\mathrm{e}\mathrm{d}\mathrm{i}\mathrm{u}\mathrm{m}\:}{\mathrm{S}\mathrm{p}.\:\mathrm{g}\mathrm{r}.\:\mathrm{o}\mathrm{f}\:\mathrm{l}\mathrm{i}\mathrm{g}\mathrm{h}\mathrm{t}\:\mathrm{m}\mathrm{i}\mathrm{n}\mathrm{e}\mathrm{r}\mathrm{a}\mathrm{l}\:-\:\mathrm{S}\mathrm{p}.\:\mathrm{g}\mathrm{r}.\:\mathrm{o}\mathrm{f}\:\mathrm{f}\mathrm{l}\mathrm{u}\mathrm{i}\mathrm{d}\:\mathrm{m}\mathrm{e}\mathrm{d}\mathrm{i}\mathrm{u}\mathrm{m}\:}$$

Based on the data in Table 2, all the heavy minerals, such as cassiterite, uranothorite, columbite, zircon, ilmenite, fluorite, and hematite, display substantially higher specific gravity and CC values compared with the light minerals, quartz and feldspar^44^. Since each valuable heavy mineral exhibits a CC value well above 1.25, gravity separation using a shaking table is expected to be highly effective for isolating these minerals from the light fraction. The results strongly support the predicted success of gravity-based beneficiation for this sample.

**Table 2:** Specific gravity, concentration criterion, and magnetic properties of principal minerals from the studied Um Naggat greisenized granite sample^44^.

Um Naggat mineral content	Specific gravity	Concentration criterion (CC)	Magnetic properties
Quartz/Feldspar	2.6	1	Diamagnetic
Muscovite	3	1.3	Paramagnetic
Fluorite	3.2	1.4	Diamagnetic
Zircon	4.7	2.3	Diamagnetic
Columbite	5.2	2.6	Paramagnetic
Ilmenite	4.7	2.3	Paramagnetic
Cassiterite	7.0	3.7	Diamagnetic
Aegirine	3.6	1.6	Paramagnetic
Almandine	4.3	2.1	Paramagnetic
Uranothorite	5.4	2.8	Paramagnetic
Hematite	5.2	2.6	Paramagnetic

The material balance data (Table 3) summarizes the performance of the gravity separation process applied to Um Naggat greisenized granite feed sample using a Wilfley shaking table operated through two successive stages, namely the rougher and the scavenger stages.The rougher stage effectively upgraded the total heavy mineral (THM) content from 1.95% in the feed to 9.95% in the rougher concentrate, representing a substantial enrichment of the heavy mineral fraction. This product accounted for 11.41 wt% of the feed and achieved a recovery of 58.22% of the total heavy minerals. The scavenger stage, applied to the rougher tailings, further recovered a portion of the heavy minerals lost during the rougher operation, producing an additional concentrate of 14.14 wt% with a grade of 3.02% THMs, corresponding to a recovery of 21.87%.

The combined concentrate from both stages represented 25.55 wt% of the feed, grading 6.11% THMs, and achieving an overall recovery of 80.09%. The final tailings fraction, constituting 74.45 wt% of the feed, contained only 0.52% THMs, accounting for 19.91% of the total heavy minerals.

Two key performance parameters further highlight the efficiency of this separation process. The enrichment ratio (C/F), was 3.13, indicating that the concentrate contained 3.13 times the THM concentration of the original feed. Additionally, the ratio of concentration (F/C), was approximately 3.9. This implies that for every 100 tons of feed, the process yielded about 25.55 tons of concentrate under the applied operating conditions.

**Fig. 13:**
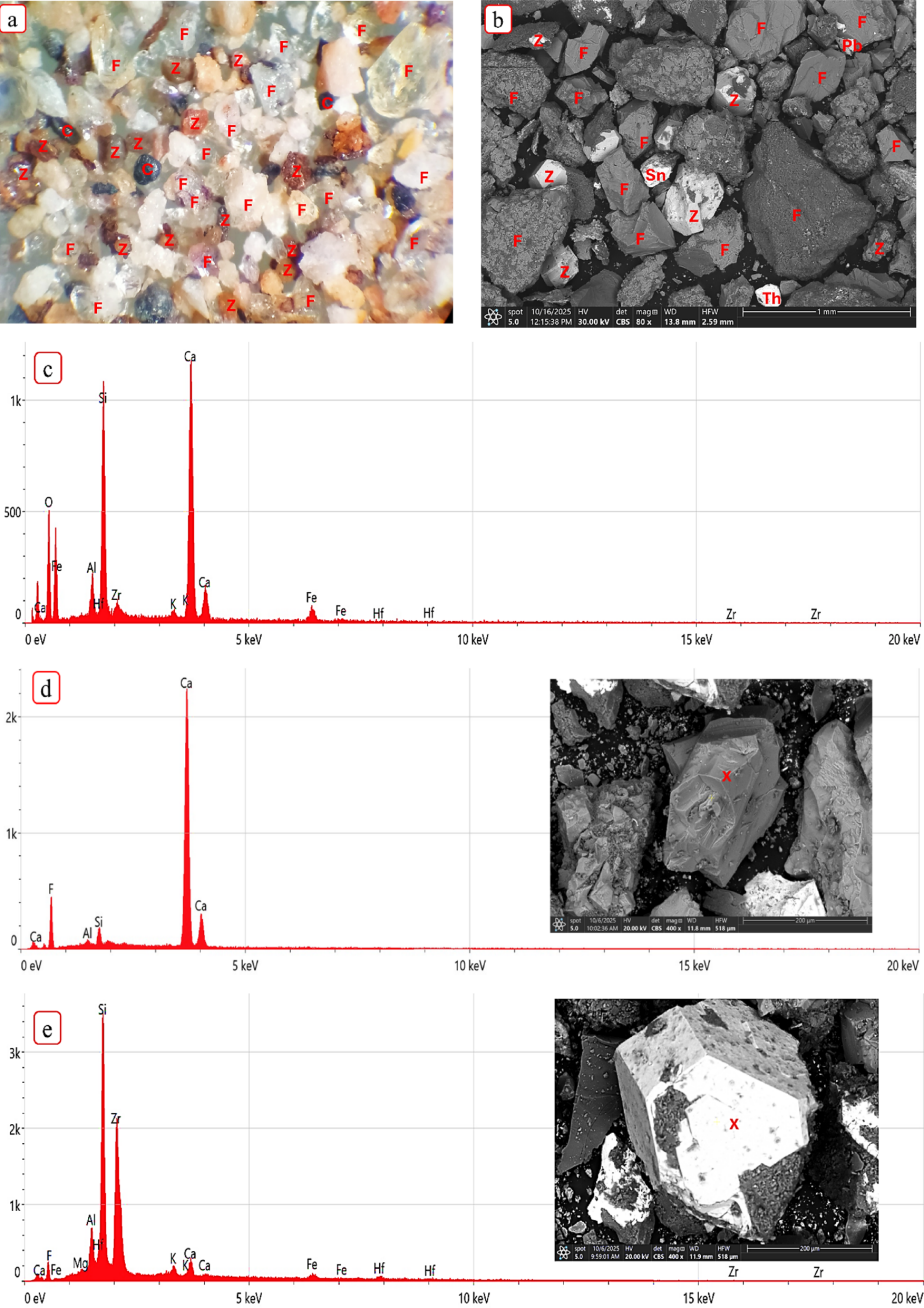

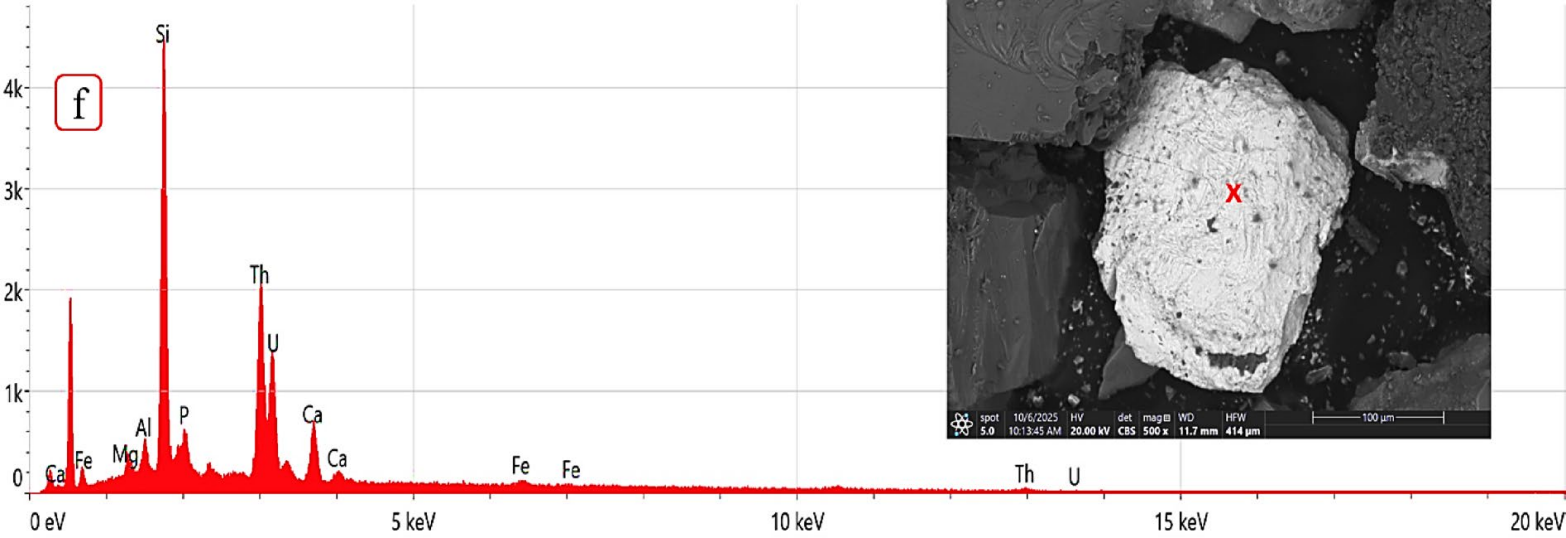
(**a**) Stereoscopic image of the heavy mineral concentrate obtained by a shaking table. (**b**) BSE image of the concentrate. (**c**) EDS spectrum of the concentrate. (**d**–**f**) BSE images with corresponding EDS spot analyses of representative grains from the concentrate, identified as fluorite (**d**), zircon (**e**), and uranothorite (**f**) from Um Naggat sample.

Overall, these results demonstrate that the two-stage shaking table separation achieved efficient beneficiation of Um Naggat greisenized granite sample, significantly enriching the heavy mineral content while maintaining high recovery. This process proved to be an effective preliminary concentration step suitable for subsequent upgrading through magnetic or flotation separation techniques. Confirmation of the mineral composition in the concentrated products, based on stereoscopic picture as well as SEM-EDS/BSE image (Fig. 13a–c), reveals the dominance of fluorite as the principal mineral phase (Fig. 13d), alongside significant enrichment of zircon (Fig. 13e) and trace of uranothorite (Fig. 13f). The presence of these key minerals in the concentrated fraction provides strong evidence of the selective liberation and successful concentration of valuable components during gravity separation, validating the efficacy of this beneficiation strategy for further mineral processing.

**Table 3:** Material balance of the gravity separation process for Um Naggat greisenized granite feed sample using a Wilfley shaking table.

Products of tabling		Yield (%)	THMs assay (%)	THMs recovery (%)
Concentrate	Roughing stage	11.41	9.95	58.22
	Scavenging stage	14.14	3.02	21.87
	Total concentrate	C = 25.55	c = 6.11	80.09
Tailing	Total	T = 74.45	t = 0.52	19.91
Feed	Total	F = 100	f = 1.95	100

#### Magnetic separation

**Fig. 14:**
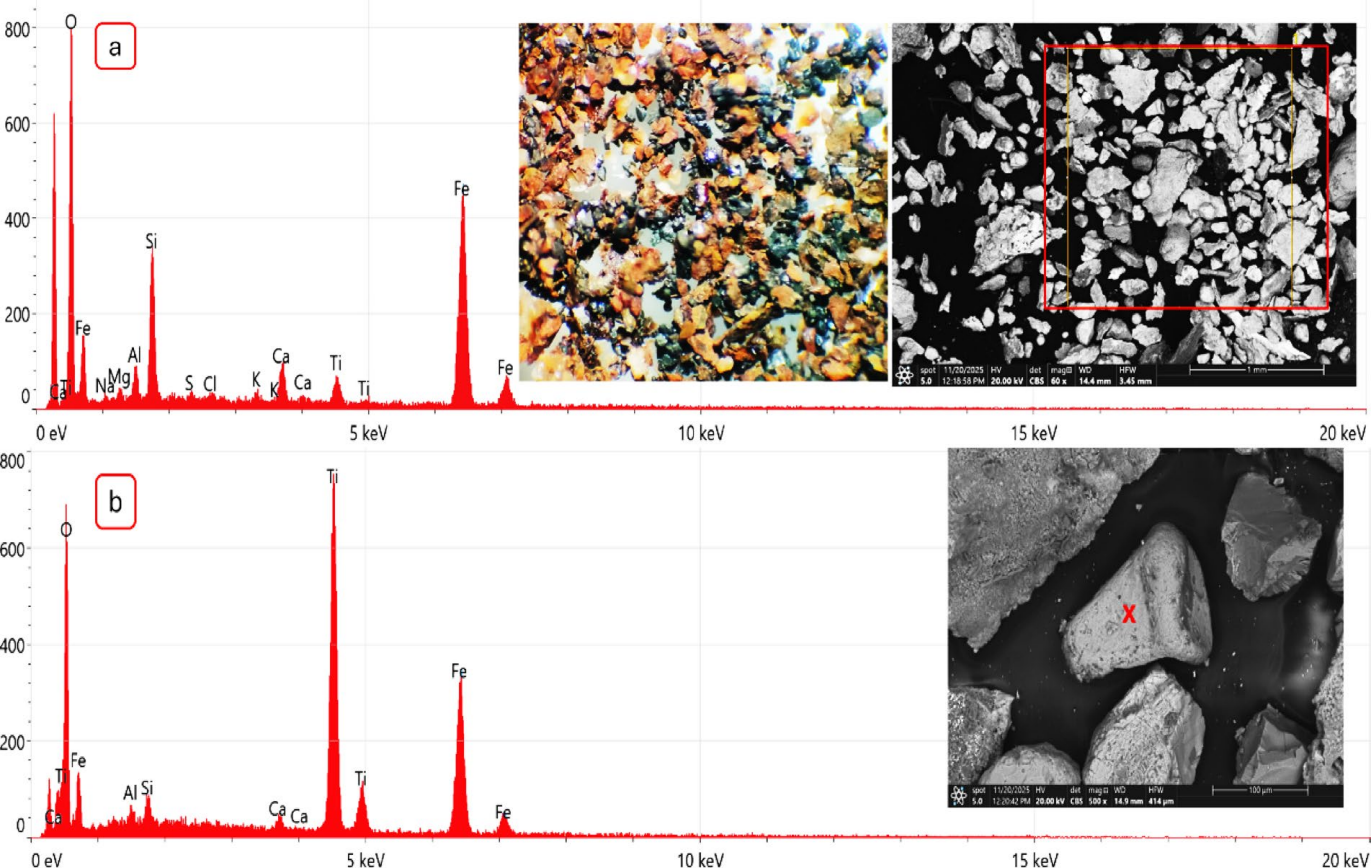
(**a**) BSE image and EDS spectra as well as stereoscopic image of magnetic fraction at 0.8 amperes. (**b**) BSE image and EDS spot analysis of an individual grain in the magnetic fraction, identifying ilmenite.

High-intensity magnetic separation (HIMS) was applied to the heavy-table concentrate derived from Um Naggat greisenized granites to evaluate the effectiveness of physical beneficiation in isolating fluorite and associated rare-metals mineralization. The process was conducted in two successive stages. In the first stage at 0.8 A, strongly magnetic minerals—mainly iron oxides and ilmenite—were efficiently removed. As shown in Fig. 14, the magnetic fraction consists predominantly of Fe–Ti oxide phases, with stereoscopic and BSE images revealing dense, dark grains of uniform morphology (Fig. 14a), while the EDS spectra display strong Fe, Ti, and O peaks. Spot analysis of an individual grain (Fig. 14b) confirms ilmenite as the principal Fe–Ti mineral, demonstrating the effectiveness of low-current separation in removing these phases prior to rare-metals concentration.

**Fig. 15:**
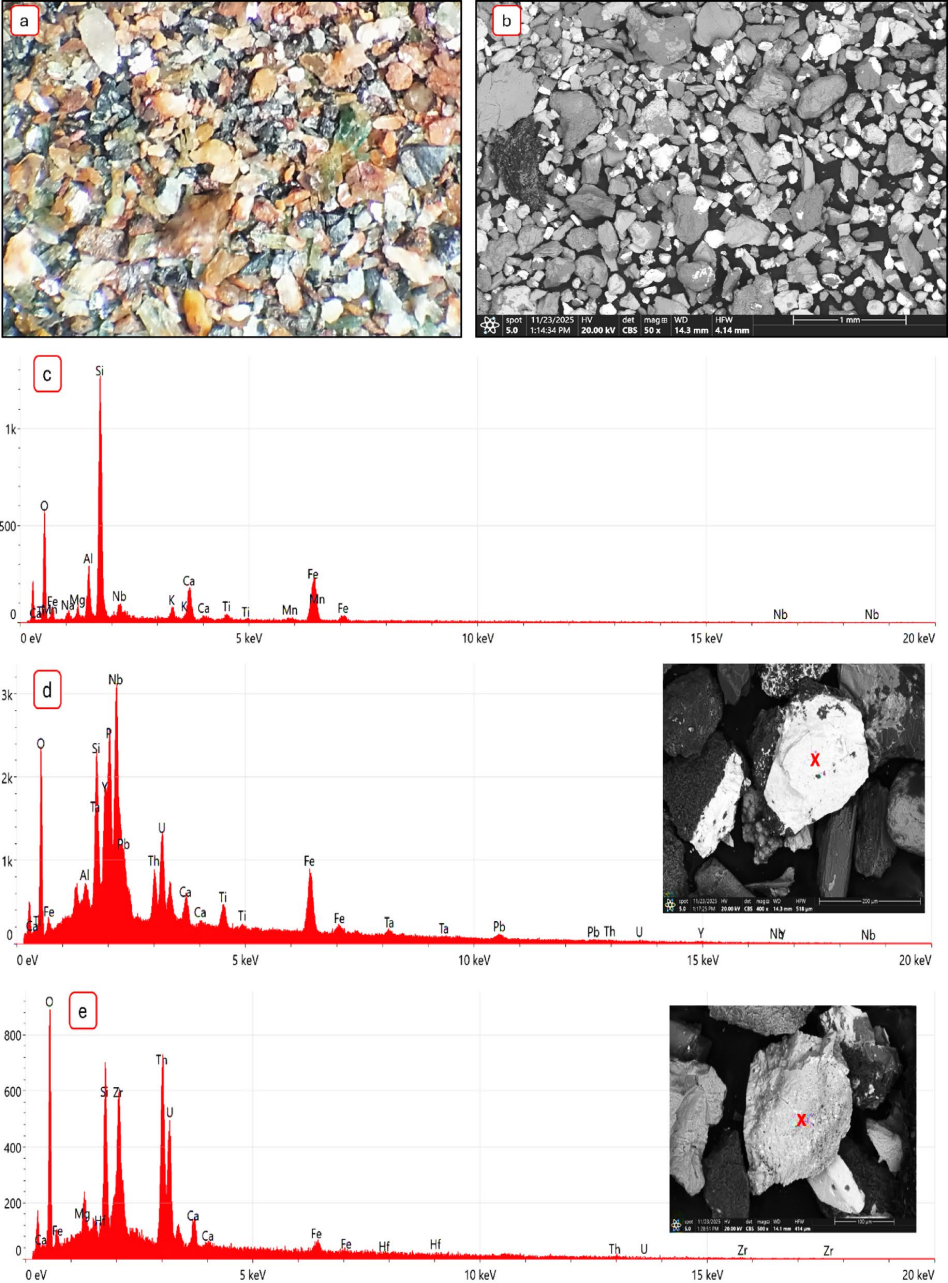

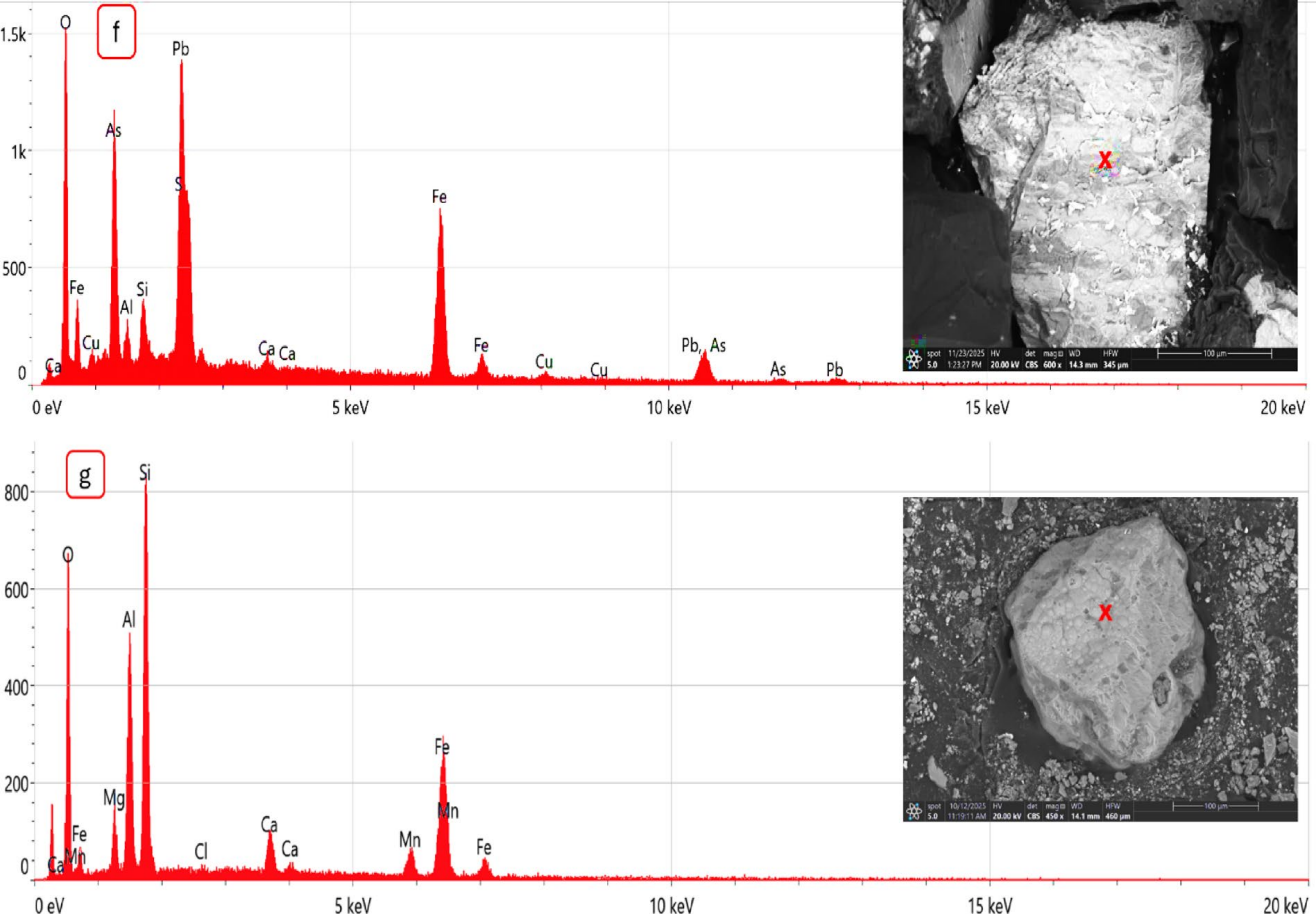
(**a**) Stereoscopic image of the magnetic fraction at 3 amperes obtained by HIMS (**b**) BSE image of the magnetic fracction. (**c**) EDS spectrum of the magnetic fracction. (**d**) BSE image and EDS spot analysis of an individual grain, identifying Nb-mineral. (**e**) BSE image and EDS spot analysis of an individual grain, identifying thorite. (**f**) BSE image and EDS spot analysis of an individual grain, identifying galena, (**g**) BSE image and EDS spot analysis of an individual grain, identifying garnet from Um Naggat sample.

The non-magnetic product from the first stage was subjected to a second separation at 3 A to distinguish paramagnetic rare-metals minerals from diamagnetic species. The magnetic fraction produced at this current intensity (illustrated in Fig. 15) exhibits a diverse assemblage dominated by Nb-, Th-, Pb-, and Fe–Mg–Ca–Al–bearing minerals, including columbite, thorite, galena, and almandine garnet. The stereoscopic and BSE images (Fig. 15a, b) show angular, dense grains typical of these mineral phases, while bulk EDS spectra (Fig. 15c) reveal elevated Fe, Ti, and Si contents. Spot EDS analyses (Fig. 15d–g) further confirm Nb-rich grains, Th-bearing thorite, Pb-bearing galena, and Fe–Mg–Ca–Al–rich garnet, highlighting the complex rare-metals mineral suite successfully concentrated through magnetic separation.

Although galena is typically considered a diamagnetic mineral, SEM-BSE and EDS analyses of the identified galena grains (Figs. 12b and 15f) revealed detectable Fe contents. The presence of Fe may reflect minor Fe-bearing inclusions, submicroscopic intergrowths, or limited chemical substitution within the galena grains. Such Fe-bearing components can locally enhance magnetic susceptibility, thereby explaining the recovery of some galena particles in the 3 A magnetic fraction.

**Fig. 16:**
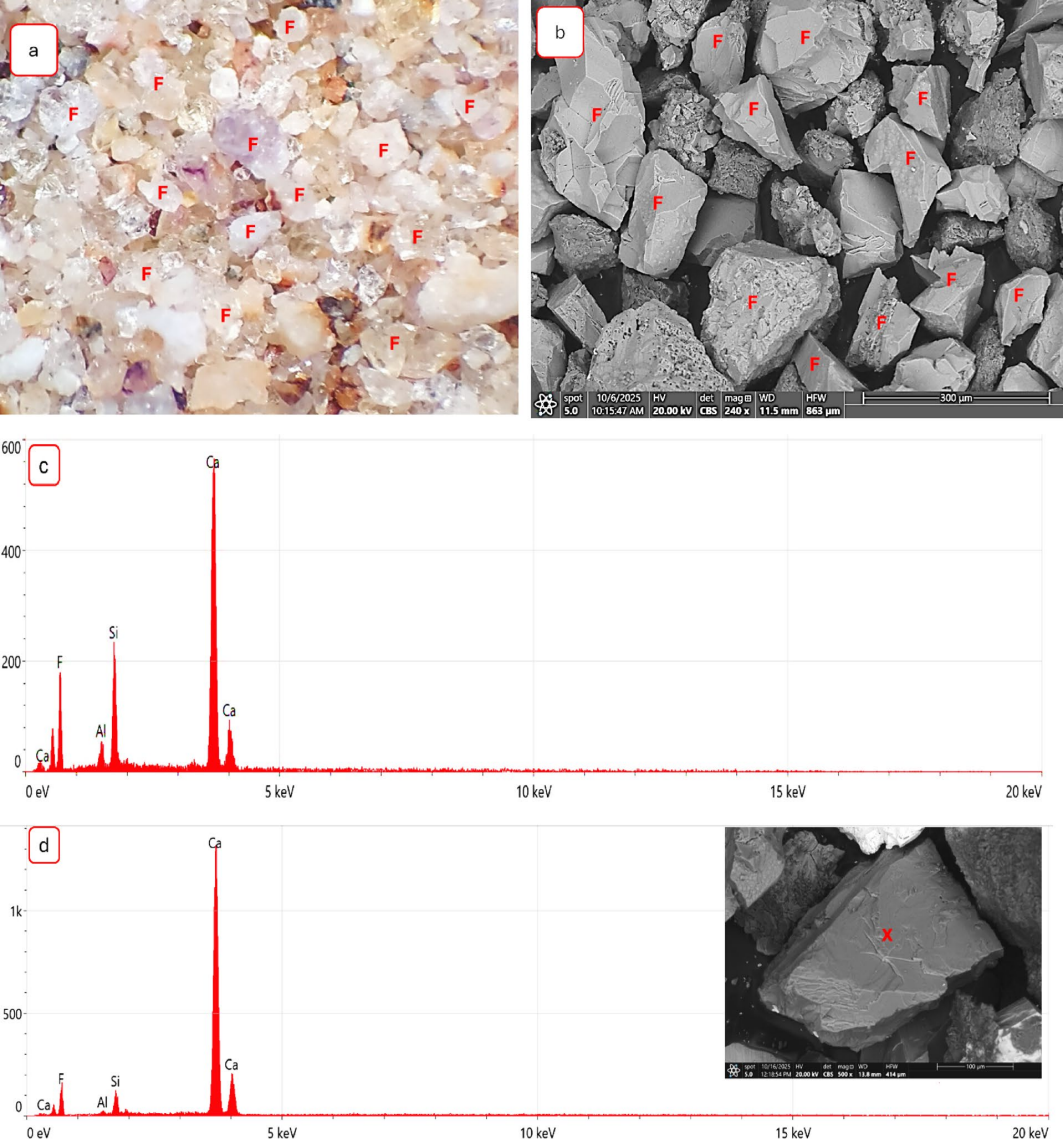

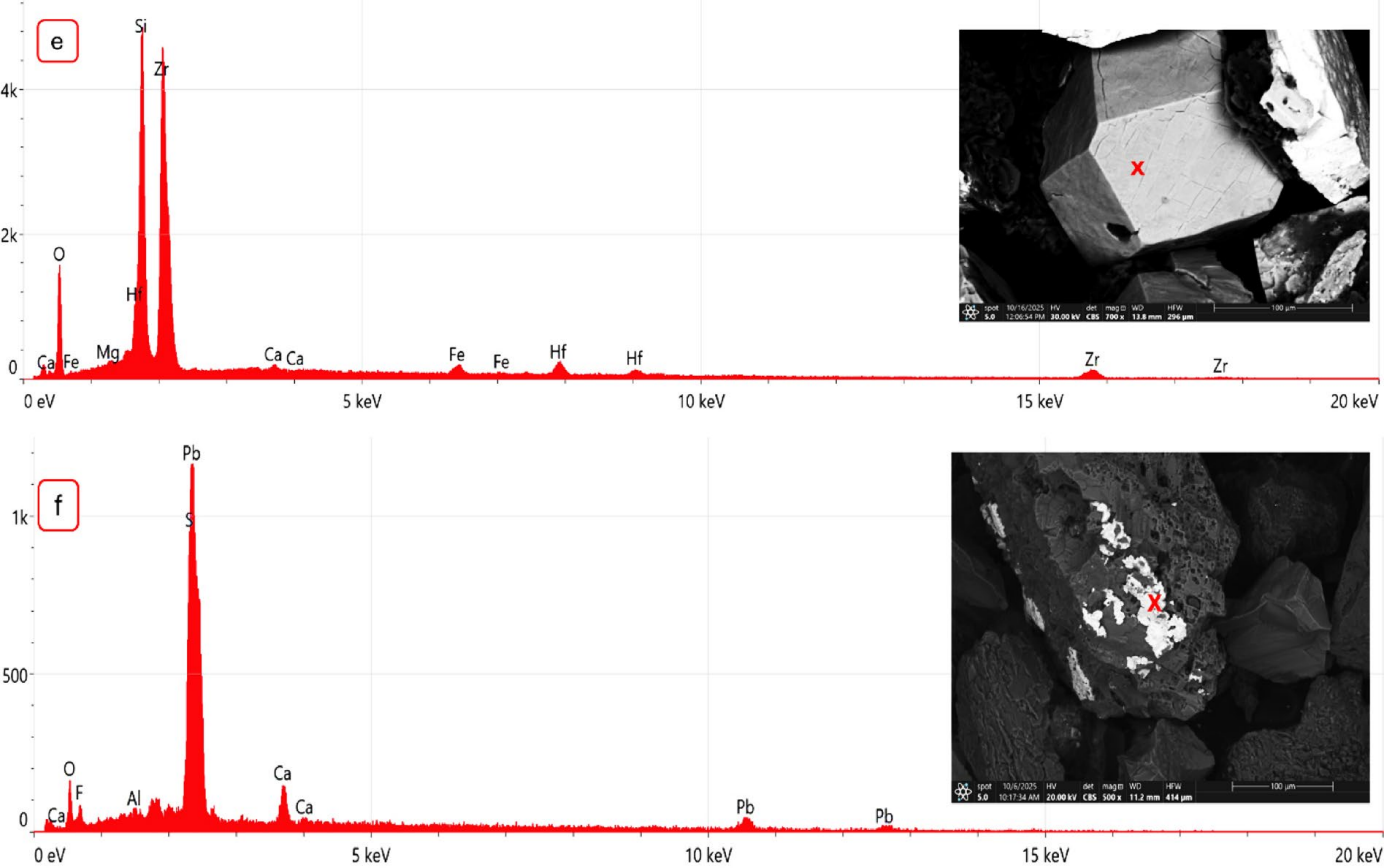
(**a**) Stereoscopic image of the non-magnetic concentrate obtained by HIMS (**b**) BSE image of the concentrate. (**c**) EDS spectrum of the concentrate. (**d**) BSE image and EDS spot analysis of an individual grain in the concentrate, identifying fluorite. (**e**) BSE image and EDS spot analysis of an individual grain in the concentrate, identifying zircon. (**f**) BSE image and EDS spot analysis of an individual grain in the concentrate, identifying galena from Um Naggat sample.

In contrast, the diamagnetic fraction obtained at 3 A (presented in Fig. 16) is overwhelmingly dominated by fluorite, accompanied by minor but economically significant traces of zircon and galena. The stereoscopic and BSE images (Fig. 16a, b) show abundant transparent to translucent fluorite grains, consistent with its non-magnetic behavior. The bulk EDS spectrum (Fig. 16c) is characterized by strong Ca and F peaks, confirming fluorite as the major constituent. Spot analyses (Fig. 16d–f) identify pure Ca–F fluorite, Zr–Si–rich zircon, and Pb-bearing galena.

Overall, the two-stage HIMS procedure successfully separates fluorite and concentrates the associated rare-metals mineralization from Um Naggat greisenized granites. These results demonstrate the clear capability of physical separation techniques to differentiate and enrich fluorite, columbite, thorite, zircon, galena, and garnet, supporting their potential for economic recovery in the studied area.

**Fig. 17:**
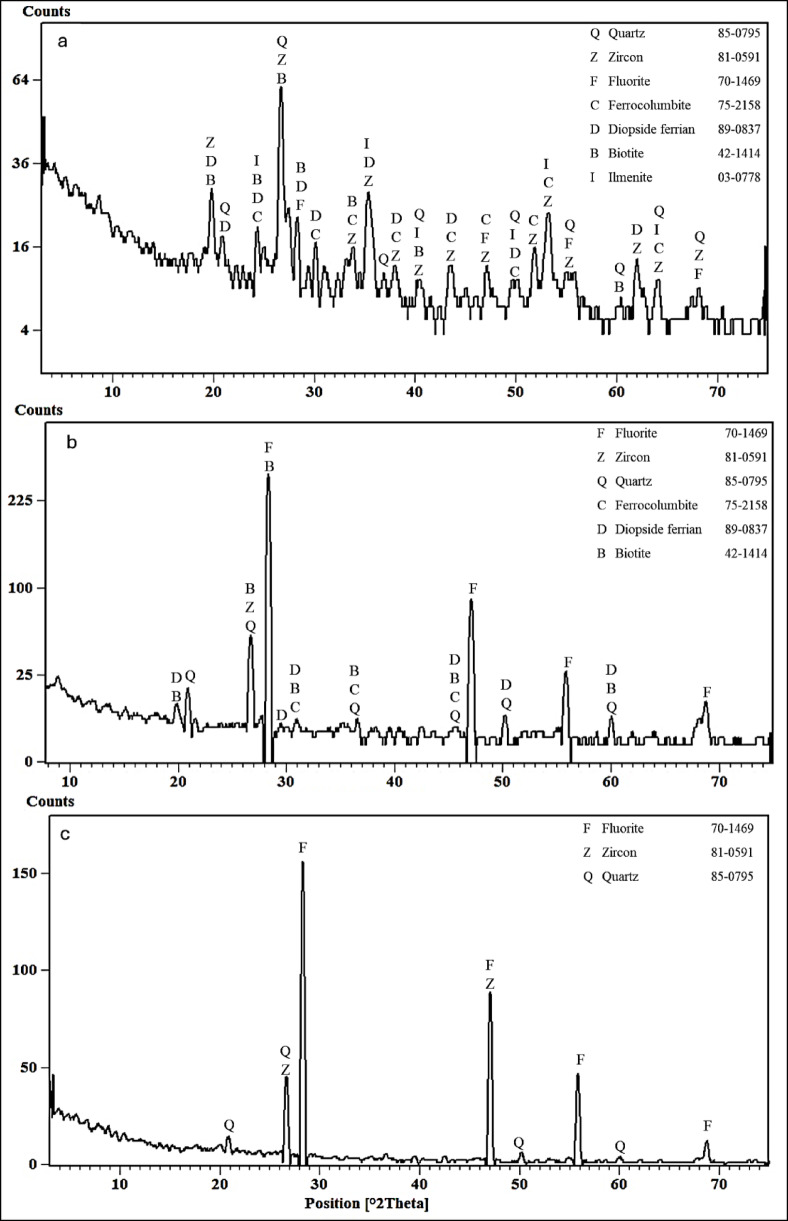
(**a**) XRD pattern of Um Naggat greisenized granite feed sample; (**b**) XRD pattern of the heavy concentrate produced by shaking-table separation; and (**c**) XRD pattern of the fluorite concentrate obtained following gravity and magnetic separation.

The XRD patterns presented in Fig. 17 demonstrate the effectiveness of the applied physical separation scheme in selectively enriching fluorite and separating it from the associated rare-metals mineralization. The feed sample from Um Naggat greisenized granite (Fig. 17a) is characterized by dominant quartz reflections (PDF 85–0795), with subordinate peaks corresponding to fluorite (PDF 70-1469), zircon (PDF 81–0591), biotite (PDF 42-1414), and ilmenite (PDF 03-0778), consistent with the complex mineralogical composition of the raw ore.

Following gravity concentration on the shaking table (Fig. 17b), quartz peaks are markedly reduced, while the reflections of higher-density minerals become more pronounced, indicating effective removal of light gangue and enrichment of fluorite, zircon, and other rare-metals-bearing phases in the heavy fraction.

Subsequent magnetic separation (Fig. 17c) produces a concentrate dominated by sharp, high-intensity fluorite peaks, with zircon, ilmenite, and other rare-metals minerals greatly diminished or absent. These results confirm that the integrated gravity–magnetic separation flowsheet efficiently isolates fluorite in a nearly pure form from the accompanying rare-metals mineralization.

## Conclusion

This study demonstrates that the greisenized granites of Um Naggat area host significant fluorite and associated rare-metals mineralization. Integrated mineralogical, geochemical, and beneficiation investigations confirmed that fluorite is the dominant mineral phase in the final concentrate, as evidenced by XRD and SEM-EDS analyses, while the occurrence of Nb-, Ta-, Zr-, Sn-, U-, Th-, and REE-bearing minerals enhances the resource potential and strategic importance of the mineralization.

The total heavy mineral (THM) fraction constitutes approximately 1.95 wt% of the bulk sample, of which fluorite represents approximately 1.47 wt% (about 75% of the THM assemblage). Gravity concentration using a Wilfley shaking table successfully upgraded the THM content from 1.95 wt% in the feed to 6.11 wt% in the combined concentrate, achieving an overall THM recovery of approximately 80.09%. Subsequent high-intensity magnetic separation effectively partitioned the heavy-mineral concentrate into fluorite-rich diamagnetic and rare-metals-bearing magnetic fractions, enabling improved mineralogical differentiation and concentration of the target mineral phases.

The laboratory-scale beneficiation results demonstrate the effectiveness of the applied gravity and magnetic separation flowsheet for upgrading fluorite and associated rare-metals minerals from the studied Um Naggat greisenized granite. However, the present work does not evaluate large-scale process performance, economic viability, or the comparative efficiency of alternative beneficiation methods. Therefore, the applicability of the proposed flowsheet should be considered within the context of the investigated material and the laboratory conditions employed.

This work provides valuable baseline information on the beneficiation behavior of fluorite–rare-metals mineralization in greisenized granites and contributes to the understanding of similar mineral systems in Egypt and elsewhere. Further investigations should focus on quantitative mineralogical characterization, detailed chemical analysis of concentrate products, optimization of gravity and magnetic separation parameters, evaluation of complementary beneficiation techniques such as flotation, pilot-scale testing, and downstream metallurgical extraction studies. Such research is necessary to improve recovery and concentrate quality and to provide a comprehensive assessment of the technical and economic feasibility of resource development.

## Data Availability

The datasets generated and analyzed during the current study are included in this manuscript.
